# Exosome-like vesicles derived from Hertwig's epithelial root sheath cells promote the regeneration of dentin-pulp tissue

**DOI:** 10.7150/thno.43156

**Published:** 2020-04-27

**Authors:** Sicheng Zhang, Yan Yang, Sixun Jia, Hong Chen, Yufeng Duan, Xuebing Li, Shikai Wang, Tao Wang, Yun Lyu, Guoqing Chen, Weidong Tian

**Affiliations:** 1 State Key Laboratory of Oral Disease, West China Hospital of Stomatology, Sichuan University, Chengdu, China;; 2 National Engineering Laboratory for Oral Regenerative Medicine, West China Hospital of Stomatology, Sichuan University, Chengdu, China;; 3 National Clinical Research Center for Oral Diseases, West China Hospital of Stomatology, Sichuan University, Chengdu, China;; 4 Department of Oral and Maxillofacial Surgery, West China Hospital of Stomatology, Sichuan University, Chengdu, China;; 5 School of Medicine, University of Electronic Science and Technology of China, Chengdu, China.

**Keywords:** epithelial-mesenchymal interaction, Hertwig's epithelial root sheath cell, exosome-like vesicle, odontogenic differentiation, pulp-dentin regeneration.

## Abstract

**Background**: The formation of dentin-pulp involves complex epithelial-mesenchymal interactions between Hertwig's epithelial root sheath cells (HERS) and dental papilla cells (DPCs). Earlier studies have identified some of the regulatory molecules participating in the crosstalk between HERS and DPCs and the formation of dentin-pulp. In the present study we focused on the role of HERS-secreted exosomes in DPCs and the formation of dentin-pulp. Specifically, we hypothesized that exosome-like vesicles (ELVs) might mediate the function of HERS and trigger lineage-specific differentiation of dental mesenchymal cells. To test our hypothesis, we evaluated the potential of ELVs derived from a HERS cell line (ELVs-H1) in inducing *in vitro* and *in vivo* differentiation of DPCs.

**Methods**: ELVs-H1 were characterized using transmission electron microscopy and dynamic light scattering. The proliferation, migration, and odontoblast differentiation of DPCs after treatment with ELVs-H1, was detected by CCK8, transwell, ALP, and mineralization assays, respectively. Real time PCR and western blotting were used to detect gene and protein expression. For *in vivo* studies, DPC cells were mixed with collagen gel combined with or without ELVs and transplanted into the renal capsule of rats or subcutaneously into nude mice. HE staining and immunostaining were used to verify the regeneration of dentin-pulp and expression of odontoblast differentiation markers.

**Results**: ELVs-H1 promoted the migration and proliferation of DPCs and also induced odontogenic differentiation and activation of Wnt/β-catenin signaling. ELVs-H1 also contributed to tube formation and neural differentiation *in vitro*. In addition, ELVs-H1 attached to the collagen gel, and were slowly released and endocytosed by DPCs, enhancing cell survival. ELVs-H1 together with DPCs triggered regeneration of dental pulp-dentin like tissue comprised of hard (reparative dentin-like tissue) and soft (blood vessels and neurons) tissue, in an *in vivo* tooth root slice model.

**Conclusion**: Our data highlighted the potential of ELVs-H1 as biomimetic tools in providing a microenvironment for specific differentiation of dental mesenchymal stem cells. From a developmental perspective, these vesicles might be considered as novel mediators facilitating the epithelial-mesenchymal crosstalk. Their instructive potency might be exploited for the regeneration of dental pulp-dentin tissues.

## Introduction

The dental pulp being responsible for the nutritional supply, dentin production, and sensation of the tooth is critical for its vitality 1. However, the dental pulp is also vulnerable to infections resulting from dental care, trauma, and multiple restorations, with such infections resulting in pulpitis and pulp necrosis. The current endodontic treatment of irreversible pulp disease, known as root canal therapy (RCT), cannot restore the function of dental pulp and thereby results in a permanently devitalized tooth that might be more susceptible to structural failure. It has been widely accepted that maintaining and regenerating the vitality of dental pulp is essential for the long-term integrity and viability of teeth. Recent advances in regenerative medicine suggested that mesenchymal stem cells, growth/differentiation factors or cytokines, and migration/homing factors, as well as biomaterials could offer hope for the full regeneration of dental pulp 2-9. However, despite the promising results in regenerating the dental pulp, these approaches have been shown to be insufficient in achieving full functional recovery of dental pulp, by still facing several challenges, including low viability, decreased regenerative potential of embedded cells, limited sources, and safety and ethical problems associated with clinical application 10-13. Therefore, exploring new strategies for the successful regeneration of functional dental pulp has become an urgent need.

The regeneration of tissues or organs through the incorporation of concepts of developmental biology has opened up a new frontier in regenerative medicine, and has been used in various tissues, such as hair follicles, salivary glands, and the gastric mucosal 14-16. During embryonic development, nearly all organ germs are known to be induced by reciprocal interactions between organ-inductive potential stem cells in the embryonic epithelium and mesenchyme 17, 18. Accordingly, regarding the formation of teeth, the interaction between dental epithelial and mesenchymal cells has also been shown to promote the morphogenesis of teeth through complex signaling networks 19, 20. Specifically, for the development of the root of the tooth, Hertwig's epithelial root sheath (HERS) derived from the inner and outer enamel epithelia has been reported to guide the proliferation, migration and differentiation of apical papilla via the epithelial-mesenchymal interaction (EMI), functioning as a signaling center to guide the formation of the root 21, 22. Noted, HERS is known to determine the size, shape, and number of tooth roots, and any disturbance in the formation of HERS might lead to malformations affecting the structure, shape, number, length, and other features of the root 23, 24.During the development of the root of the tooth, various secreted molecules and growth factors involved in sonic hedgehog (SHH), Wnt/β-catenin, and BMP signaling have been reported to mediate interactions between dental papilla cells (DPC) and HERS 19. Thus, HERS cells might be an attractive cell source in the field of regeneration of tooth root tissue.

Exosomes are known as a family of nanoparticles with a diameter in the range of 40-150 nm produced by multivesicular bodies, carrying bioactive cytokines, growth factors, signaling lipids, and RNA, and acting as novel mediators in the communications or interactions between cells during processes of organogenesis or pathogenesis 25-27. Due to their important roles in intercellular communications, exosomes have lately gained increased attention as a novel bioactive structure that could be used in therapeutic applications and tissue regeneration 28. However, because of limited selective markers, current protocols for isolation of exosomes have been shown to yield a mixture of vesicles with similar size and morphology to exosomes, which are often defined as exosome-like vesicles (ELVs) 29, 30. Recently, ELVs were confirmed in the epithelium-mesenchyme crosstalk in the development of teeth and it was further reported that exosome vesicles could induce the regeneration of the dental pulp through enhanced odontogenesis 31 32. However, nearly all previous studies using ELVs to generate dental pulp tissue focused on the dental mesenchyme 32, 33, while ignoring the important role of dental epithelium-derived ELVs in the epithelium-mesenchyme crosstalk.

As an important structure of the dental epithelium, HERS is believed to function as a developmental center, with the interactions between HERS and the dental mesenchyme, such as dental papilla, and dental follicle, playing an important role in the formation of the root of the tooth and periodontal tissue 21, 22. However, HERS is known to be a transient structure assembled in the early period of the elongation of the root 34, thus making it difficult to obtain enough primary cells for research. To address this limitation, our lab has established an immortalized HERS cell line named HERS-H1 35, 36. This cell line was demonstrated to be similar in morphology and characteristics to primary HERS cells, and could also induce DPCs-derived odontogenic differentiation 35, 36. This HERS-H1 line provided a new way for abundantly and stably cultivating HERS cells *in vitro*, and also made it possible to obtain large-scale engineered dental epithelial ELVs that could be applied in the regeneration of the dental pulp tissue. In this study, we found that HERS-H1-derived ELVs (ELVs-H1) mediated dental epithelial-mesenchymal interactions and provided the possibility of using ELVs-H1 as biomimetic tools in generating engineered pulp-dentin tissue.

## Materials and methods

### Animals

Animals were obtained from Dashuo Experimental Animal Co. Ltd. (Chengdu, China). All animals were maintained under standardized conditions at 21 °C and a 12 h light cycle, with free access to food and water. This study was reviewed and approved by the Ethics Committees of the State Key Laboratory of Oral Diseases, West China School of Stomatology, Sichuan University.

### Cell culture

The rat HERS cell line used in this study was established in our previous study 35. HERS-H1 cells were cultured in epithelial medium (ScienCell, USA) supplemented with 1% penicillin/streptomycin. To collect the culture medium for isolation of ELVs, HERS-H1 cells were cultured to 80% confluence, washed twice with PBS, then switched to medium with exosome-depleted FBS (2%, SBI, USA) and cultured for another 2 d. Dental papilla cells (DPCs) were isolated from unerupted first molars of 1 to 3-d-old postnatal SD rats as previously described 37. To inhibit the activity of the Wnt/β-catenin pathway, dickkopf WNT signaling pathway inhibitor 1 (DKK1) (100 ng/mL, R&D systems, USA) was used for 2 h to induce the phosphorylated degradation of β-catenin. Cells were grown in a humidified atmosphere at 37 °C with 5 % CO_2_, and the medium was changed every 3 d.

### Treatment of HERS-H1 cells and coculture with dental papilla cells

First, DPCs cells were seeded in standard 12-well plates. Concomitantly, HERS-H1 cells were labeled with the DiO green fluorescent dye (Life tech, USA) according to the manufacturer's instructions and seeded in the upper compartment of a transwell plate (0.4 μm). Noted, HERS-H1 cells were pretreated with either the GW4869 inhibitor of exosome biogenesis (10 μM, Selleck, USA) or vehicle for 24 h before being cocultured with DPCs cells. Accordingly, DPCs cells were cocultured with HERS-H1 cells for 24 h and then harvested and counterstained with DAPI (Life Technologies, USA) for nucleus and phallotoxins (Cell Signaling Technology, USA) for cytoskeleton. Images were captured using a confocal microscope (Olympus FV1200, Olympus, Japan). For the analysis of the interaction between HERS-H1 and DPCs cells, the coculture system was incubated for 3 d. Total cellular proteins of DPCs cells were obtained using the Total Protein Extraction Kit (KeyGEN, China), and differentiation markers were determined by western blotting.

### Isolation and identification of exosome-like vesicles

Exosome-like vesicles (ELVs) derived from HERS-H1 cells (ELVs-H1) were isolated as previously described 38. First, the culture medium was collected and centrifuged at 2000 *g* for 30 min, and then the supernatant was introduced into Amicon Ultra-15 Centrifugal Filter Units with Ultracel-100K (100 000 Mw cutoff membrane, Millipore, USA) and centrifuged at 5000 *g* for 30 min. Subsequently, ELVs from the culture medium were isolated using the Total Exosome Isolation TM reagent (Life Technologies, USA) following the manufacturer's protocol. Pellets were resuspended in 100 μL PBS and the concentration of protein was determined using the BCA method. All procedures were performed at 4 °C. Isolated ELVs were stored at -80 °C or immediately used in experiments.

The ultrastructure of ELVs was analyzed under a transmission electron microscope (Hitachi H7500, Japan). Representative markers of ELVs, such as tumor susceptibility 101 (Tsg101), CD63 molecule (CD63), and CD9 were detected using western blot analysis. To determine the size of purified ELVs, dynamic light scattering measurement was performed using the Zetasizer Nano ZS90 system (Malvern, UK).

### Experiments of uptake of exosome-like vesicles

Isolated ELVs were labeled with the DiO green fluorescent dye according to the manufacturer's instructions. DiO-labeled ELVs were suspended with exosome-depleted medium and added to the medium of DPCs cells for 2, 24, and 48 h, respectively. After that, DPCs cells were washed twice with PBS, fixed in 4% paraformaldehyde, and stained with DAPI. Fluorescence signals were captured with a confocal microscope (Olympus FV1200, Olympus). All experiments were performed at least in triplicate.

### Cell proliferation and migration assays

For the following assay, DPCs cells were seeded in 96-well plates at 2 × 10^4^ cells per well and incubated overnight. Then, DPCs were maintained in medium containing ELVs-H1 of 0, 80, 160, and 240 µg/mL, respectively for 5 d. The proliferation of DPCs cells was analyzed using the Cell Counting Kit-8 (Dojindo, Japan) according to the manufacturer's instructions. The migration of DPCs cells was analyzed using a Chemotaxicell Chamber (8 µm, Osaka, Japan). Briefly, DPCs cells were seeded into the upper chamber at a density of 10^5^ cells per well, and ELVs (0, 80, 160, and 240 µg/mL) were added to the bottom wells and incubated for 12 h. Subsequently, DPCs cells migrated to the lower surface of the membrane were fixed with 4% paraformaldehyde and stained using Giemsa staining solution (Solarbio, China). Images were captured using an inverted microscope (Olympus). Cells were counted and analyzed using the Image J software. All experiments were performed at least in triplicate.

### Alkaline phosphatase assay

For the alkaline phosphatase assay, DPCs were cultured in medium or osteogenic medium (OM, consisting of basal medium, 0.01 μM dexamethasone, 50 μg/mL ascorbic acid, 0.01 μM dihydroxyvitamin-D3, and 10 mM glycerophosphate) with or without ELVs-H1. At day 3, ALP activity was analyzed with the ALP kit (Jiancheng, China) and normalized on the basis of equivalent protein concentrations. The absorbance of each well was measured at 520 nm using the Multiskan Go Spectrophotometer (Thermo Fisher Scientific). All experiments were performed at least in triplicate.

### *In vitro* mineralization assay

Respectively, DPCs were seeded in a 12 well plate (2 × 10^5^ per well) and cultured with osteogenic medium with or without ELVs-H1. After 5 and 7 days, cells were fixed with 4% paraformaldehyde, washed and stained with 0.1% Alizarin red S (Sigma-Aldrich, St Louis, MO, USA) for 30 min. Mineralized bone nodules were destained with 10% cetylpyridinium chloride, and the concentration of calcium was analyzed by measuring absorbance at 562 nm. At least 3 technical replicates were performed.

### Renal capsule transplantation

To characterize the effect of ELVs-H1 on the odontogenic differentiation of DPC cells *in vivo*, DPC cells were mixed with Matrigel (Corning, USA) at a density of 10^8^ cells/mL, with or without ELVs-H1 (2 mg/mL). Cell-matrigel pellets (5 × 10^5^, 5 μL) were then transplanted into the left renal capsule of each 8-week-old SD rat. Six rats were randomly divided into 2 groups: a control and a DPCs+ELVs-H1 group. Each group contained at least 3 replicates. Grafts were obtained after 4 weeks, fixed with 4% paraformaldehyde, decalcified with buffered 10% EDTA, and then embedded in paraffin for further experiments.

### Tube formation assays

Human umbilical vein endothelial cells (HUVECs) were pretreated with or without ELVs-H1 (80 μg/mL) for 3 d, and then cells were collected and seeded onto matrigel-coated 96-wells plates at a density of 10^4^ cells per well. After 4 h incubation, images were captured using an inverted microscope (Olympus). The total number of nodes, meshes, and junctions of all tubing within each field were measured using the Image J software. At least 3 technical replicates were performed.

### Neural differentiation assay

For neural differentiation, DPCs (10^5^ cells) were seeded in a 15 mm cell culture dish and incubated with control or neurogenic medium (NM, consisting of Neurobasal A Media, 100 U/mL penicillin, B27 supplement, 100 μg/mL streptomycin, 20 ng/mL epidermal growth factor, and 40 ng/mL basic fibroblast growth factor) with or without ELVs-H1 (80 μg/mL) for 3 d. Cells were fixed and then analyzed using cell immunofluorescence. Images were captured with a confocal microscope. At least 3 technical replicates were performed.

### Characterization of hydrogel combined with exosome-like vesicles

Hydrogel samples combined with ELVs were prepared by resuspending ELVs-H1 with Cellmatrix Type I-P collagen gel (Nitta Gelatin, Japan), and then rapidly incubated at 37 °C for 30 min.

For analysis using scanning electron microscopy (SEM), samples were harvested, fixed with 2.5% glutaraldehyde at 4 °C, dehydrated and dried in a critical point dryer, and finally observed under a SEM (Inspect F, FEI, Netherlands). The experiment was repeated at least 3 times. For analysis of fluorescence, collagen gels were mixed with a suspension of DiO-labeled ELVs, incubated at 37 °C for 24 h, fixed with 4% paraformaldehyde, and washed extensively with PBS. Images were examined under a fluorescence confocal microscope (Olympus FV1200, Olympus).

To detect ELVs released from the hydrogel, hydrogels with different concentrations of ELVs (0, 2, 4, and 6 mg/mL) were incubated in PBS for 7 d. Consecutively, PBS was changed every day and the protein concentration of the obtained PBS was measured using the BCA method. The amount released per day was calculated as total proteins minus the control. At least 3 technical replicates were performed.

### Analysis of the uptake of exosome-like vesicles in collagen gel

For this assay, DPCs (1 × 10^4^) combined with DiO-labeled ELVs-H1 (80 μg/mL) were seeded on a collagen gel. Cells were cultured for 2, 24, and 48 h, washed with PBS, fixed with 4 % paraformaldehyde, and then stained with phallotoxins and DAPI. Images were obtained using a confocal microscope. At least 3 replicates were performed.

### Live/dead assay

For the live/dead assay, DPCs were mixed with ELVs-H1 (80 μg/mL) and collagen gel, whereas cells only suspended with collagen gel were used as the control. After incubation for 1, 3, and 5 d, the adhesion and viability of DPCs were examined under a confocal microscope (Olympus FV1200, Olympus) by staining the cells with the live/dead assay kit (keyGEN BioTECH) using double staining of calcein AM and propidium iodide. Accordingly, the cytoplasm of viable cells was stained green by calcein AM, whereas that of non-viable cells was stained red by propidium iodide. The total number of live and dead cells within each field was measured using the Image J software. At least 3 technical replicates were performed.

### Subcutaneous transplantation in the tooth root slice model

For this experiment, DPCs cells (10^8^ cells/mL) were mixed with collagen gel and combined with or without ELVs-H1 (2 mg/mL), and then used to fill the root canal spaces of a root slice. The treated dentin matrix (TDM) canal was obtained from extracted incisors of pigs and formed to an internal diameter of 2 mm and a height of 3 mm as previously described 39. Samples were subcutaneously implanted on the right and left back of nude mice. Six mice were randomly designated into 2 groups for the *in vivo* transplantation experiments: a collagen gel+ DPCs and a collagen gel+ ELVs+ DPCs group. The first molars of 4-week-old rats were used as a native control. Grafts were obtained at 4 weeks postoperatively, fixed with 4% paraformaldehyde, decalcified with 10 % buffered EDTA, and then embedded in paraffin for further experiments.

### Hematoxylin/eosin and immunohistochemical staining

For histology and immunohistochemistry analysis, sections of 6 µm were made for hematoxylin/eosin staining. Immunohistochemical staining was performed as previously described 35. Primary anti-DSPP (1/200; Zen Bioscience), anti-DMP1 (1/200; Santa Cruz Biotechnology), and anti-OCN (1/200; Zen Bioscience) antibodies were used. Detection of crossreacted secondary antibodies was performed using the DAB kit (Gene Tech, China). Accordingly, quantitative analysis of the staining was conducted by measuring the average optical density using the ImageJ software. At least 3 serial sections from each sample were examined, and at least 3 samples were included in each group.

### Immunofluorescent staining

Tissue sections or cells were fixed, washed and blocked, and then incubated with the following primary antibodies: anti-Nestin (1/200; Abcam), anti-NF200 (1/200; Abcam), anti-MBP101 (1/200; Abcam), anti-Active β-catenin (1/200; Cell Signaling Technology), anti-DSPP (1/200; Zen Bioscience), anti-DMP1 (1/200; Santa Cruz Biotechnology), anti-CD31 (1/200; Zen Bioscience), and anti-VEGF (1/200; Abcam). The goat anti-rabbit 488 (1/200; Invitrogen) and goat anti mouse 488 (1/200; Invitrogen) secondary antibodies were used. Sections or cells were counterstained with DAPI, mounted with fluorescent mounting media and visualized using a fluorescence confocal microscope (Olympus FV1200, Olympus). Accordingly, quantitative analysis of the staining was conducted by measuring the average optical density using the ImageJ software. At least 3 replicates were performed.

### Real-time PCR

For analysis of the mRNA expression, DPCs were cultured in 6-well plates and stimulated with ELVs-H1 for 24 h. The RNAiso Plus (Takara, Japan) was used to extract total RNA according to the manufacturer's protocol. Respectively, 2 μg of RNA were reverse transcribed to cDNA using the Revert Aid First Strand cDNA Synthesis Kit (Thermo Scientific). Real-time RT-PCR was conducted using the SYBR Premix Ex Taq (TaKaRa Biotechnology). The reaction was performed using the QuantStudio 6 Flex system (Applied Biosystems, Foster City, CA, USA). The following primers were used in this study: AXIN2: 5ʹ-TAACCCCTCAGAGCGATGGA-3ʹ and 5ʹ-CCTCCTCTCTTTTACAGCAGGG-3ʹ, TCF7: 5ʹ-CCAAGAATCCACCACAGGAGG-3ʹ and 5ʹ-GCCTAGAGCACTGTCATCGG-3ʹ, GAPDH: 5ʹ-TATGACTCTACCCACGGCAAG-3ʹ and 5ʹ-TACTCAGCACCAGCATCACC-3ʹ. All performed procedures followed the manufacturer's protocol. All experiments were performed in triplicates and repeated 3 times.

### LC-MS/MS analysis and bioinformatic analysis

ELVs-H1 were lysed with lysis buffer which containing 100 mM NH_4_HCO_3_ (pH 8), 6 M Urea and 0.2% SDS, followed by 5 min of ultrasonication on ice. The lysate was centrifuged at 12000 *g* for 15 min at 4℃. The supernatant was collected and quantified with a BCA Protein Assay Kit (Beyotime, Shanghai, China). 120 μg of each protein sample was taken and the volume was made up to 100 μL with lysis buffer, 3 μL of 1 μg/μL trypsin and 500 μL of 100 mM TEAB buffer were added, sample was mixed and digested at 37 °C overnight. Equal volume of 1% formic acid was mixed with digested sample and centrifuged at 12000 *g* for 5 min at room temperature. The supernatant was slowly loaded to the C18 desalting column, washed with 1 mL of washing solution (0.1% formic acid, 4% acetonitrile) 3 times, then eluted twice by 0.4 mL of elution buffer (0.1% formic acid, 75% acetonitrile). The eluents were combined and lyophilized. For LC-MS/MS analysis, the separated peptides were analyzed byQ Exactive HF mass spectrometer (Thermo Fisher), with ion source of Nanospray Flex™ (ESI), spray voltage of 2.3 kVand ion transport capillary temperature of 320 °C. The resulting spectra from each fraction were searched separately against UniProtKB/Swiss-Prot database (2017_11; 556,196 entries) restricted to Rattus norvegicus (8091 sequences) by the search engines: Proteome Discoverer 2.2 (PD 2.2, Thermo). The search parameters are set as follows: mass tolerance for precursor ion was 10 ppm and mass tolerance for product ion was 0.02 Da. Carbamidomethyl was specified in PD 2.2 as fixed modifications. Oxidation of methionine (M) and acetylation of the N-terminus were specified in PD 2.2 as variable modifications. A maximum of 2 missed cleavage sites were allowed. The identified protein contains at least 1 unique peptide with FDR no more than 1.0%. Proteins containing similar peptides that could not be distinguished by MS/MS analysis were identified as a same protein group. Gene Ontology (GO) analysis and KEGG (Kyoto Encyclopedia of Genes and Genomes) were analyzed by DAVID 40, 41.

### Western blot analysis

For western blot analysis, cell pellets or ELVs were lysed with RIPA buffer containing a complete cocktail of protease inhibitors (Millipore Calbiochem, USA). Proteins were quantified using the BCA Protein Assay (BioRad, USA) and equal amounts (20 μg) of protein lysates from each sample were separated by SDS-polyacrylamide gel electrophoresis (SDS-PAGE). Separated proteins were then transferred onto polyvinylidene fluoride (PVDF) membranes. Membranes were blocked with 5 % skim milk and then incubated with the following primary antibodies: anti-Tsg101 (1/1000; Zen Bioscience), anti-CD63 (1/1000; Zen Bioscience), anti-CD9 (1/1000; Zen Bioscience), anti-HSP70 (1/1000; Zen Bioscience), anti-actin (1/5000; Abcam), anti-DSPP (1/1000; Zen Bioscience), anti-DMP1 (1/1000; Santa Cruz Biotechnology), anti-ALP (1/1000; Abcam), anti-RUNX2 (1/1000, ab76956; Abcam), anti-Active-β-Catenin (1/1000; Cell Signaling Technology), anti-Wnt3a (1/1000; Zen Bioscience), and anti-GAPDH (1/5000; Zen Bioscience). Membranes were then thoroughly rinsed and incubated with species-matched HRP-conjugated secondary antibodies. Protein bands were visualized using the Amersham ECL Select western blotting detection reagent (GE Healthcare Life Sciences, USA) in accordance with the manufacturer's protocol. Blot images were captured on an ImageQuant LAS 4000 mini system (GE Healthcare Life Sciences) and quantified by densitometric scanning (Image Quant TL; GE Healthcare Life Sciences). Experiments were performed independently for each sample, and at least 3 technical replicates were performed for each of the treated samples and controls.

### Statistical analysis

All data were expressed as mean ± standard deviation (SD). Statistical significance was assessed by using the Student's *t*-test for 2 groups. P < 0.05 was considered as statistically significant.

## Results

### HERS cell-derived exosome-like vesicles transported to dental papilla cells triggered odontoblastic differentiation

**Figure 1:**
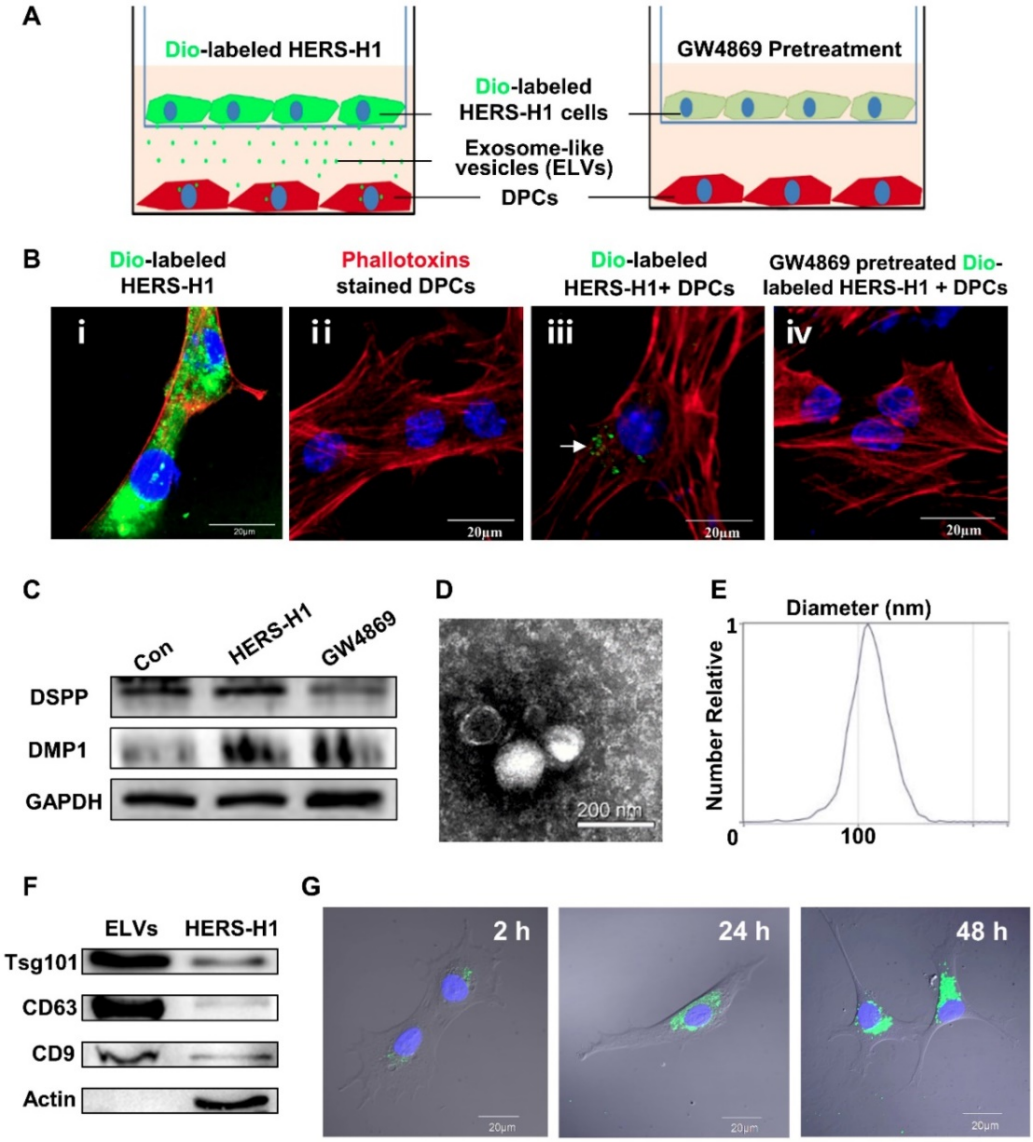
** Exosomal vesicles mediate interaction of HERS and DPCs cells** (A) Schematic diagram showing transwell coculture of HERS-H1 and DPCs cells. (B) HERS-H1 cells were labeled by DiO (B i), DPCs cells were stained with phallotoxins, and nuclei were stained with DAPI (B ii). DPCs cells cocultured with HERS-H1 cells endocytosed exosomal vesicles (white arrow) released from HERS-H1 cells (B iii), whereas DPCs cells cocultured with GW4869-pretreated HERS-H1 cells did not show the endocytosed exosomal vesicles (B iv). (C) HERS-H1 cells upregulated the expression of DSPP and DMP1 in DPCs cells, which was attenuated by pretreatment with GW4869. (D) TEM analysis of ELVs. (E) DLS showed the particle size distribution of ELVs-H1. (F) Western blot analysis of the surface markers of ELVs. (G) DPCs cells were incubated with DiO-labeled ELVs (green) for 2, 24, and 48 h, respectively. Nuclei of DPCs cells were stained with DAPI (blue). (TEM: transmission electron microscope; DLS: dynamic light scattering). Scale bars are shown. *p < 0.05 vs. Con; #p < 0.05 vs HERS-H1.

To investigate whether the transfer of ELVs occurs from HERS-H1 to DPCs cells, we used a cell coculture system as shown in Figure 1A. The HERS-H1 cells were labeled with the DiO green fluorescent dye (Figure 1B i), which labeled both the membranes of cells and ELVs 38. After coculture for 24 h, ELVs secreted from HERS-H1 cells were uptaken by DPCs cells (Figure 1B iii). HERS-H1 cells pretreated with GW4869 exhibited a decreased release of ELVs, resulting in the decreased uptake of ELVs by DPCs cells (Figure 1B iv). Western blot analysis showed that the expression of dentin matrix acidic phosphoprotein 1 (DMP1) and dentin sialophosphoprotein (DSPP) was upregulated in DPCs cells when they were cocultured with HERS-H1 cells, whereas in the case of coculture with HERS-H1 cells pretreated with GW4869 the expression of DMP1 and DSPP in DPCs cells was shown to be unaffected (Figure 1C, Figure S1A). All these results indicated that HERS cells were able to at least partly induce odontoblastic differentiation through the transport of extracellular vesicles to DPC cells.

Subsequently, we collected the conditioned medium of HERS-H1 cells and isolated extracellular vesicles. Observation under TEM revealed that ELVs-H1 appeared to be double membrane cup-like structures with an average diameter of 110 nm (Figure 1D-E). The presence of the Tsg101 protein related to exosome biogenesis, as well as the CD9 and CD63 exosomal markers were validated using western blotting, whereas the actin cellular protein was detected exclusively in the HERS-H1 cell lysate (Figure 1F). These data suggested that the vesicles in our study could be qualified as exosome-like vesicles as they were shown to possess the characteristics of exosomes 42.

### Endocytosis of HERS cell-derived exosome-like vesicles by dental papilla cells

To explore whether ELVs-H1 could be internalized, DPCs cells were cocultured with DiO-labeled ELVs-H1 for 2, 24, and 48 h respectively, and then visualized using confocal microscopy. Results showed that assembled DiO-labeled ELVs-H1 (green; Figure 1G) surrounded the nuclei after entering cells, indicating that ELVs-H1 were internalized by DPCs cells over time.

### HERS cell-derived exosome-like vesicles promoted proliferation and migration of dental papilla cells

**Figure 2:**
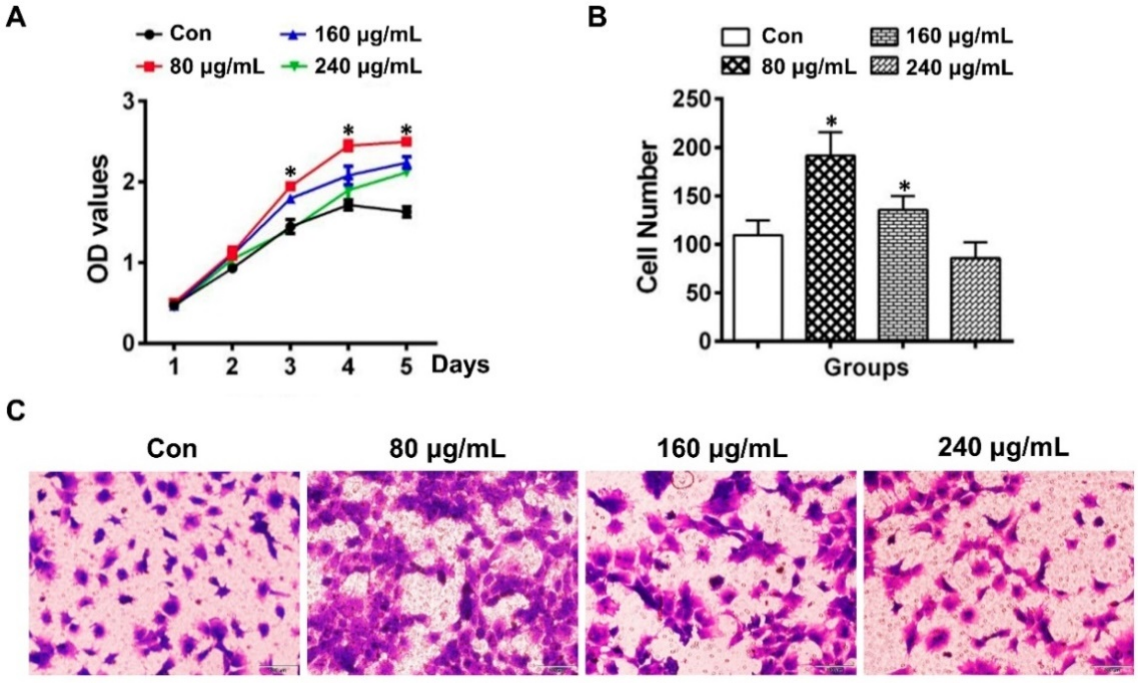
** HERS-H1 cells-derived ELVs enhanced proliferation and migration of DPCs cells.** (A) The proliferation of DPCs measured by the CCK8 assay. (B) Migrated cells per field of view from 4 different experiments. (C) Representative images of the capacity for cell migration shown by the transwell test. Scale bars represent 100 μm. *p < 0.05 vs. Con.

The proliferation of DPCs cells determined by the CCK-8 assay was demonstrated to be increased in the group of 80 μg/mL ELVs-H1, whereas the 160 and 240 μg/mL groups showed relatively lower capacity for proliferation compared with the 80 μg/mL ELVs-H1 group (Figure 2A). At the concentration of 80 μg/mL, ELVs-H1 were noted to lead to a significantly increased number of migrated DPCs cells (Figure 2B-C, p < 0.05 vs. Con), which was nearly doubled compared with the control. However, treatment with higher concentrations of ELVs-H1 (160 or 240 μg/mL), resulted to a decreased migration capacity of DPCs cells compared with the 80 μg/mL group.

### HERS cell-derived exosome-like vesicles induced odontogenic differentiation *in vitro* and *in vivo*

**Figure 3:**
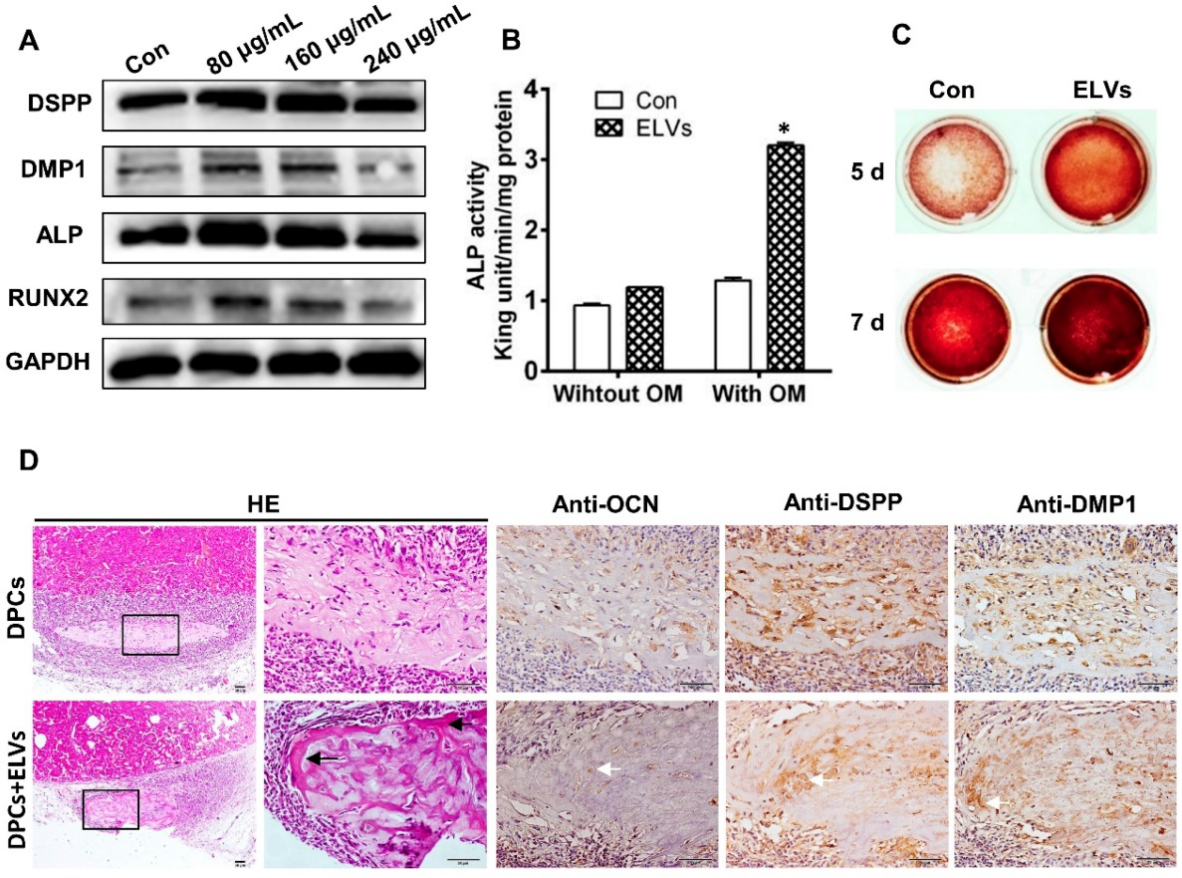
** HERS-H1 cells-derived ELVs enhanced odontogenic differentiation of DPC cells *in vitro* and *in vivo*.** (A) Western blot analysis showing the significantly upregulated expression of odontoblastic markers (DSPP, DMP1, ALP, and RUNX2) in DPC cells after treatment with various concentrations of ELVs for 3 d. (B) ALP activity assay showing that treatment with ELVs increased the alkaline phosphatase activity of DPC cells with or without OM induction. (C) Representative images of Alizarin Red S staining showing that treatment with ELVs increased the odontogenic differentiation of DPC cells. (D) H&E staining showing that mineralized tissue was generated in the DPCs+ELVs group (black arrow). Immunohistochemical staining showing the upregulated expression of odontoblastic markers (OCN, DSPP, and DMP1) in the DPCs+ELVs group (white arrow). (OM: osteogenic medium; DSPP: dentin sialophosphoprotein; DMP1: dentin matrix protein 1; ALP: alkaline phosphatase; RUNX2: runt related transcription factor 2). Scale bars represent 50 μm. *p < 0.05 vs. Con.

To investigate the effect of ELVs-H1 on odontogentic differentiation, DPCs cells were treated with different concentration of ELVs-H1 for 3 d. Total cellular proteins of DPCs cells were obtained and western blot analysis showed that ELVs-H1 at 80 μg/mL significantly upregulated the expression of DSPP, DMP1, ALP, and runt related family transcription factor 2 (RUNX2) (Figure 3A, Figure S1B, p < 0.05 vs. Con). Likewise, DPCs cells treated with osteogenic medium with ELVs-H1 (80 μg/mL) were shown to also increase the activity of alkaline phosphatase and formation of mineralized nodules *in vitro* (Figure 3B-C; Figure S1C). Furthermore, after 4 weeks of transplantation of renal capsules *in vivo*, osteoid-like structures were observed in the DPCs+ ELVs group, which were also shown to highly express osteocalcin (OCN), DSPP, and DMP1 (Figure 3D, Figure S2A). In contrast, little obvious formation of osteoid-like structures and less OCN, DSPP, or DMP1 was detected in the control group.

### HERS cell-derived exosome-like vesicles promoted tube formation and neurogenic differentiation *in vitro*

**Figure 4:**
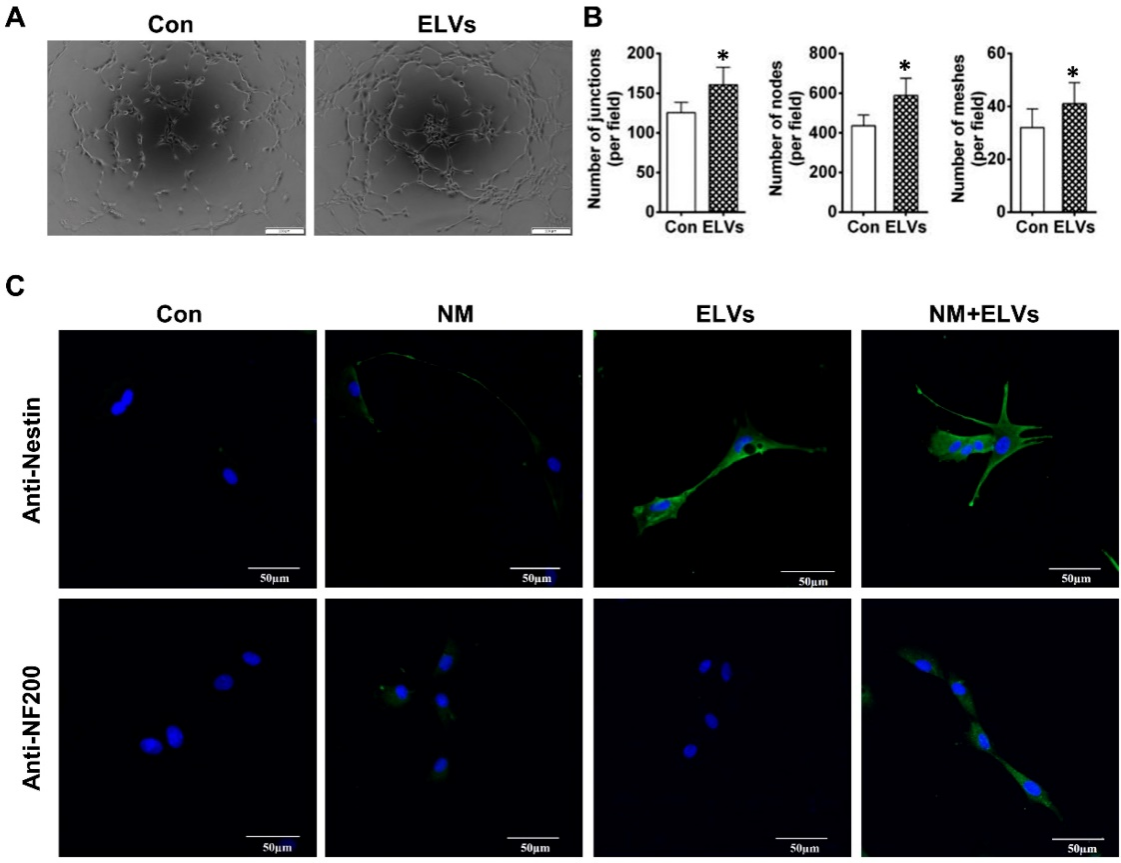
** HERS-H1 cells-derived ELVs enhanced tube formation and neurogenic differentiation *in vitro*.** (A and B) *In vitro* tube formation of HUVECs and total number of nodes, meshes, and junctions of all tubing upregulated after treatment with ELVs (80 μg/mL). (C) Representative immunofluorescence images showing the increased expression of neurogenic differentiation markers (nestin and NF200) after treatment with ELVs (80 μg/mL) for 3 d. (NM: neurogenic medium). Scale bars are shown. *p < 0.05 vs. Con.

As shown in Figure 4A, ELVs-H1 (80 μg/mL) markedly enhanced tube formation of HUVECs compared with the control. The total number of nodes, meshes, and junctions in the ELVs group were obviously higher than that in the control (Figure 4B).

The ability of ELVs-H1 in inducing enhanced neurogenic differentiation of DPCs cells was investigated by evaluating the differentiation ratios of neuronal lineage cells of DPCs cells cultured in the presence of neurogenic medium with or without ELVs-H1 (80 μg/mL). Results showed that DPCs cells treated with ELVs-H1 obviously expressed the nestin neuronal marker (Figure 4C). Under neurogenic culture conditions, DPCs cells were revealed to display changes in cellular morphology following neurogenic differentiation, including formation of axons, and dendrite-like cytoplasmic projections of cytoplasm. Cells treated with ELVs-H1 were further shown to exhibit an increased expression of the nestin and NF200 neuronal markers (Figure 4C).

### Activation of the Wnt/β-catenin signaling by exosomal Wnt3a

**Figure 5:**
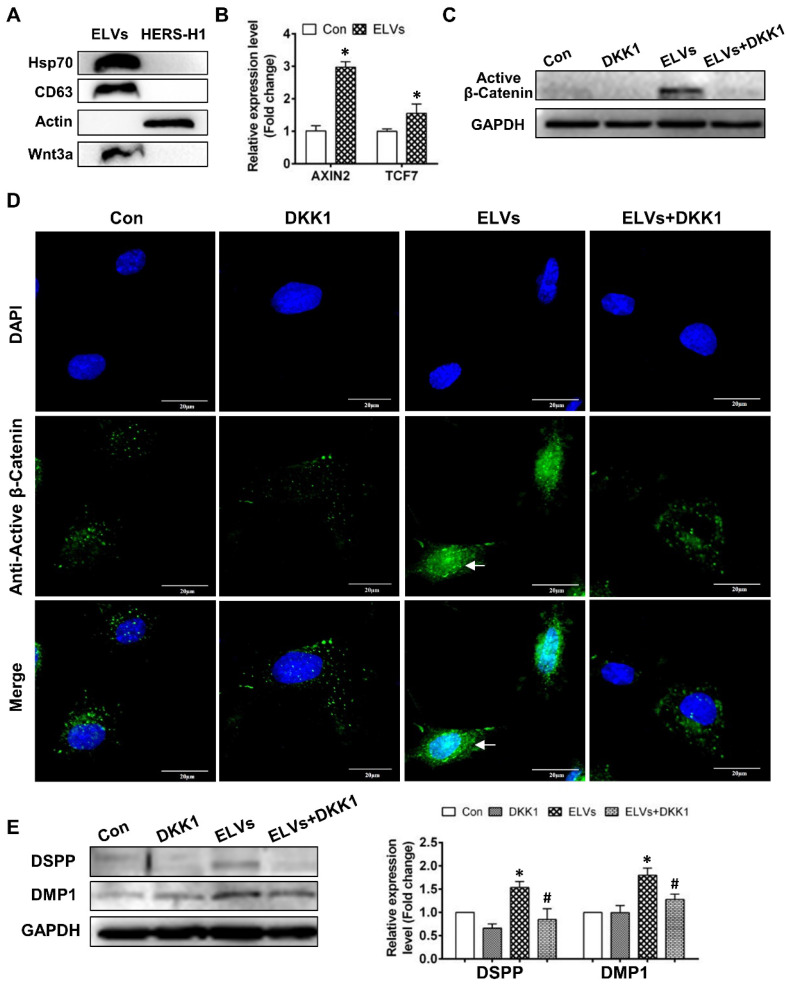
** HERS-H1 cells-derived ELVs activated Wnt/β-Catenin signaling.** (A) Immunoblots of exosomal (Hsp70, CD63) and cytoplasmatic cell (actin) markers; Wnt3a proteins in ELVs are presented in the panel. (B) Real time RT-PCR showing the upregulated expression of AXIN2 and TCF7 in DPC cells after treatment with ELVs (80 μg/mL). (C) Western blotting revealing the upregulated expression of β-catenin in DPC cells after treatment with ELVs (80 μg/mL), whereas the same marker was significantly downregulated in the ELVs+DKK1 and DKK1 groups. (D) Immunofluorescence staining of β-catenin in DPC cells after treatment with ELVs (80 μg/mL) alone or combined with DKK1. In the control, β-catenin mostly existed in the cytosol of DPC cells, even after addition of DKK1. ELVs induced the transference of β-catenin from the cytosol into the nucleus (white arrow). Accordingly, addition of DKK1 into ELVs could inhibit the transference of β-catenin from the cytosol into the nucleus. (E) Treatment with ELVs (80 μg/mL) upregulated the expression of DMP1 and DSPP, which was attenuated by treatment with DKK1. (DSPP: dentin sialophosphoprotein; DMP1: dentin matrix protein 1). Scale bars are shown. *p < 0.05 vs. Con; #p < 0.05 vs ELVs.

To comprehensively understand the characteristics of ELVs-H1, we generated proteomics data for ELVs-H1 by a label free proteomics quantification approach. In total, 204 proteins were identified in the ELVs-H1 and the identified proteins information were listed in Table S1. To gain insight into the functional roles of the proteins that were identified in ELVs-H1, we employed GO-based biological process clustering (Table S2) and KEGG pathway mapping (Table S3). Enrichment of molecules involved in regulation of cell migration, ECM-receptor interaction, PI3K-Akt signaling pathway and focal adhesion were observed. Among the identified proteins in ELVs-H1, the Wnt protein is known to be important in regulating the development of the root, and β-catenin-mediated Wnt signaling has been reported to be required in the regulation of the cranial neural crest (CNC)-derived odontoblast differentiation 43. Using western blot analysis, we confirmed that Wnt3a could be enriched in ELVs-H1 (Figure 5A), and DPCs cells treated with ELVs-H1 (80 μg/mL) were noted to exhibit a significantly increased expression of Wnt/β-catenin signaling target genes, such as axis inhibition protein 2 (AXIN2) and transcription factor 7 (TCF7) 45 (Figure 5B, p < 0.05 vs. Con). We subsequently evaluated the activity of β-catenin in DPCs cells, and immunofluorescent staining and western blot analysis confirmed that treatment with ELVs-H1 led to the markedly increased expression and nuclear translocation of β-catenin in DPCs cells (Figure 5C-D). In addition, treatment with DKK1 was shown to abrogate the upregulation and nuclear translocation of β-catenin induced by ELVs-H1 (Figure 5C-D). Moreover, treatment with ELVs-H1 was also demonstrated to result in the upregulated expression of DMP1 and DSPP, which was attenuated by treatment of DPCs cells with DKK1 (Figure 5E).

### HERS cell-derived exosome-like vesicles binding to collagen gel

**Figure 6:**
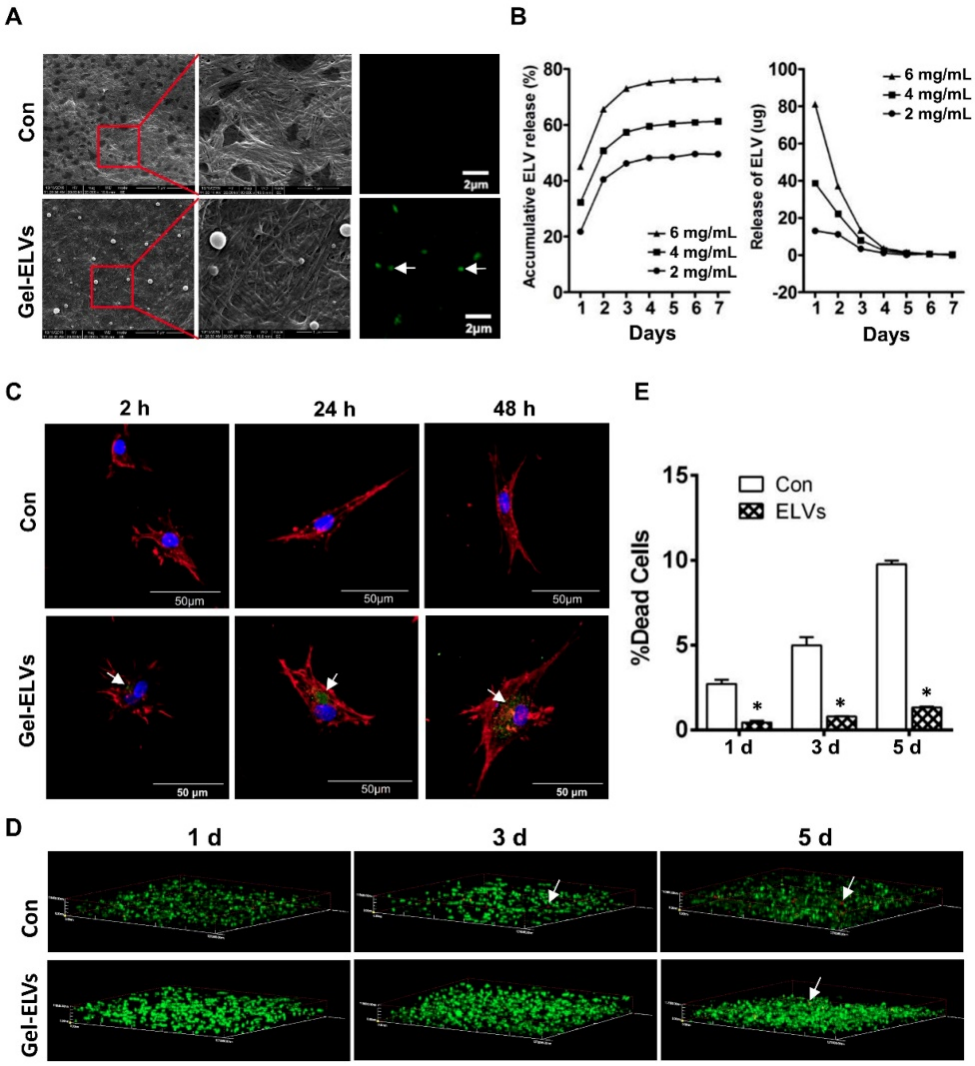
** HERS-H1 cells-derived ELVs incorporated collagen gel enhanced cell survival.** (A) ELVs within collagen gel were processed for SEM and confocal microscopy (white arrow). (B) The release efficiency of ELVs within the collagen gel was analyzed using the BCA method. (C) DPC cells endocytosed ELVs released from the collagen gel (white arrow). DPC cells were incubated onto collagen gel filled with DiO-labeled ELVs (green) for 2, 24, and 48 h, respectively. Cells were stained with phallotoxins (red) and nuclei were stained with DAPI (blue). (D) Live/dead staining of cultured DPC cells within the ELVs-containing collagen gel. Live cells are labeled with green, whereas red staining indicates dead cells. (E) Death rates of DPC cells within the collagen gel are shown. Scale bars are shown. *p < 0.05 vs. Con.

When ELVs-H1 were incubated with collagen gel, we were able to observe the binding of ELVs-H1 to the collagen gel. A representative SEM micrograph presented in Figure 6A confirmed this interaction. Respectively, ELVs-H1 were shown to adhere to the collagen gel and exhibit a regular round shape with an approximate diameter of 100 nm (Figure 6A). Confocal microscopy also revealed the presence of DiO-labeled ELVs-H1 on the collagen gel (Figure 6A).

The binding and releasing efficiency of ELVs-H1 were then quantitatively analyzed. Results presented in Figure 6B indicated a dose-dependent binding to the collagen gel. The total released amount and the release efficiency per day were demonstrated to be positively correlated with the initial density of ELVs-H1. In addition, these results indicated that ELVs-H1 tended to be sustained-released by the collagen gel in a time-dependent manner, with a continuous release for 4-6 d.

### Dental papilla cells endocytosed HERS cell-derived exosome-like vesicles released from collagen gel

To investigate whether ELVs-H1 released from collagen gel could be internalized by DPC cells, DPCs cells were seeded on the collagen gel combined with DiO-labeled ELVs-H1, followed by cultivation for 2, 24, and 48 h. Results showed that ELVs-H1 released from collagen gel were internalized by DPCs cells on a time-dependent manner (Figure 6C).

### Collagen gel incorporated with HERS cell-derived exosome-like vesicles enhanced the survival of dental papilla cells

To investigate the effect on their survival, DPCs cells were mixed with collagen gel with or without the incorporation of ELVs-H1, and then these generated constructs were cultured for 1, 3, and 5 d. Results from the live/dead assay revealed that DPCs cells adhered into the collagen gel, with most of them remaining viable. In contrast, the number of dead cells in the control group from day 1 to day 5 was observed to be significantly more relative to that of the ELVs group (Figure 6D-E, Table S4).

### HERS cell-derived exosome-like vesicles enhanced *in vivo* formation of pulp-dentin complex

**Figure 7:**
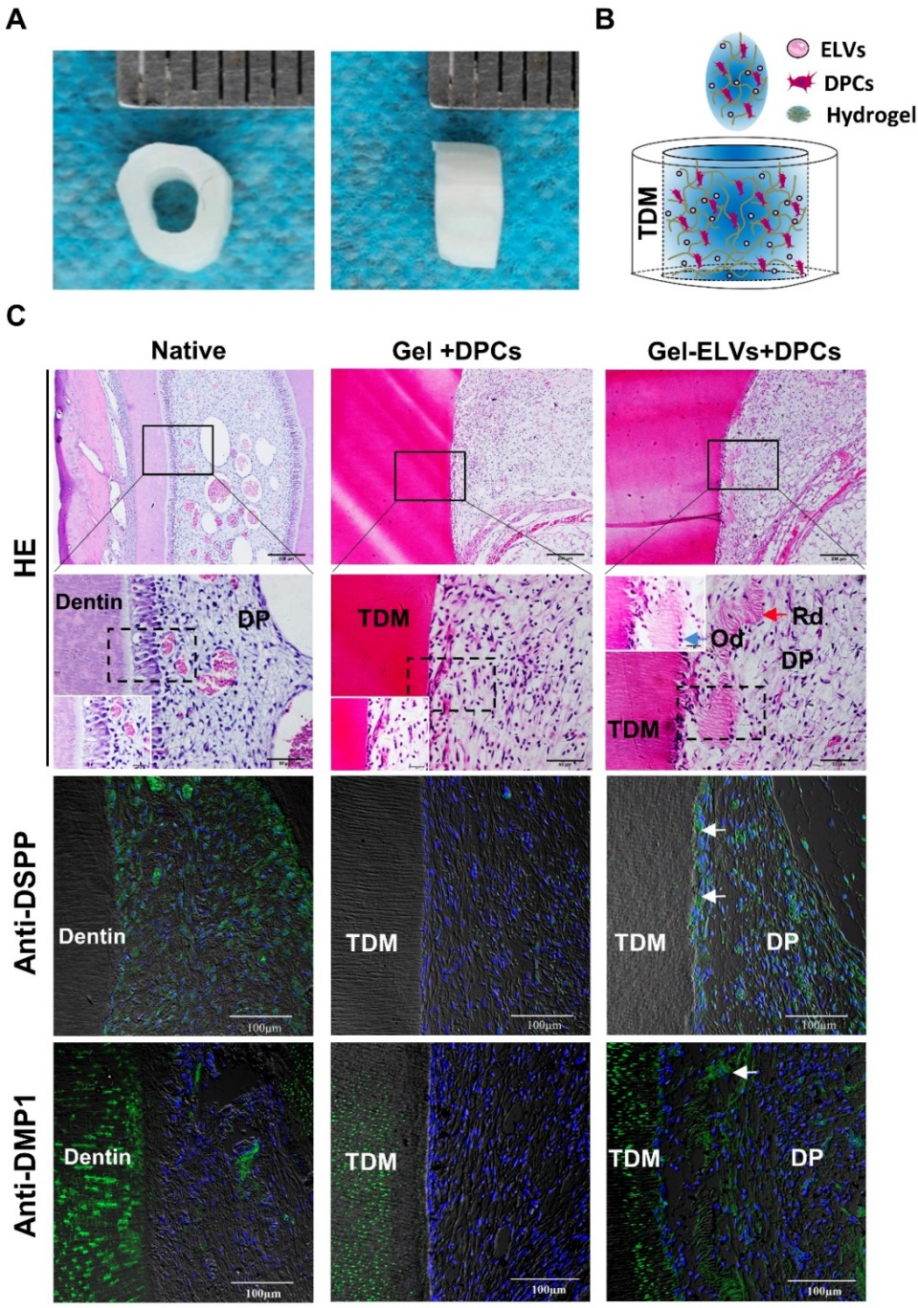
** HERS-H1 cells-derived ELVs increased *in vivo* formation of pulp-dentin complex.** (A) The implanted tooth root slice, with an internal diameter of 2 mm and a height of 3 mm. (B) Schematic of the preparation of *in vivo* transplants. DPC cells were resuspended with collagen gel mixed with ELVs, and then injected into TDM tubes. (C) HE staining showing odontoblast-like cells and regenerated dentin-like tissue (red arrows) at the interface between the dentin and pulp-like tissue. Immunofluorescence analysis showing the upregulated expression of odontogenic differentiation markers (DSPP and DMP1) in the Gel-ELVs+DPCs group, with positive staining (white arrows) represented by green stains. (DSPP: dentin sialophosphoprotein; DMP1: dentin matrix protein 1; TDM: treated dentin matrix; Rd: regenerated dentin-like tissue; Od: odontoblast-like cell; DP: dental pulp-like tissue). Scale bars are shown.

In the Gel-ELVs+DPCs group, we could observe the formation of newly regenerated structures, such as predentin-like tissue, polarizing odontoblast-like cells, and collagen fibers, resembling those of the pulp-dentin tissue of the normal tooth group (Figure 7C). However, the Gel+DPCs group was only shown to exhibit an extensive deposition of extracellular matrix and formation of collagen. Immunofluorescence observations revealed an increased expression of DSPP, DMP1, and β-catenin at the interface between the dentin and soft tissue in the Gel-ELVs+DPCs group (Figure 7C, Figure S2B-C). These results indicated that the ELVs-H1-incorporated collagen gel triggered the increased expression of odontogenic and Wnt signaling molecular markers, and induced odontogenic differentiation in the tooth root slice model.

**Figure 8:**
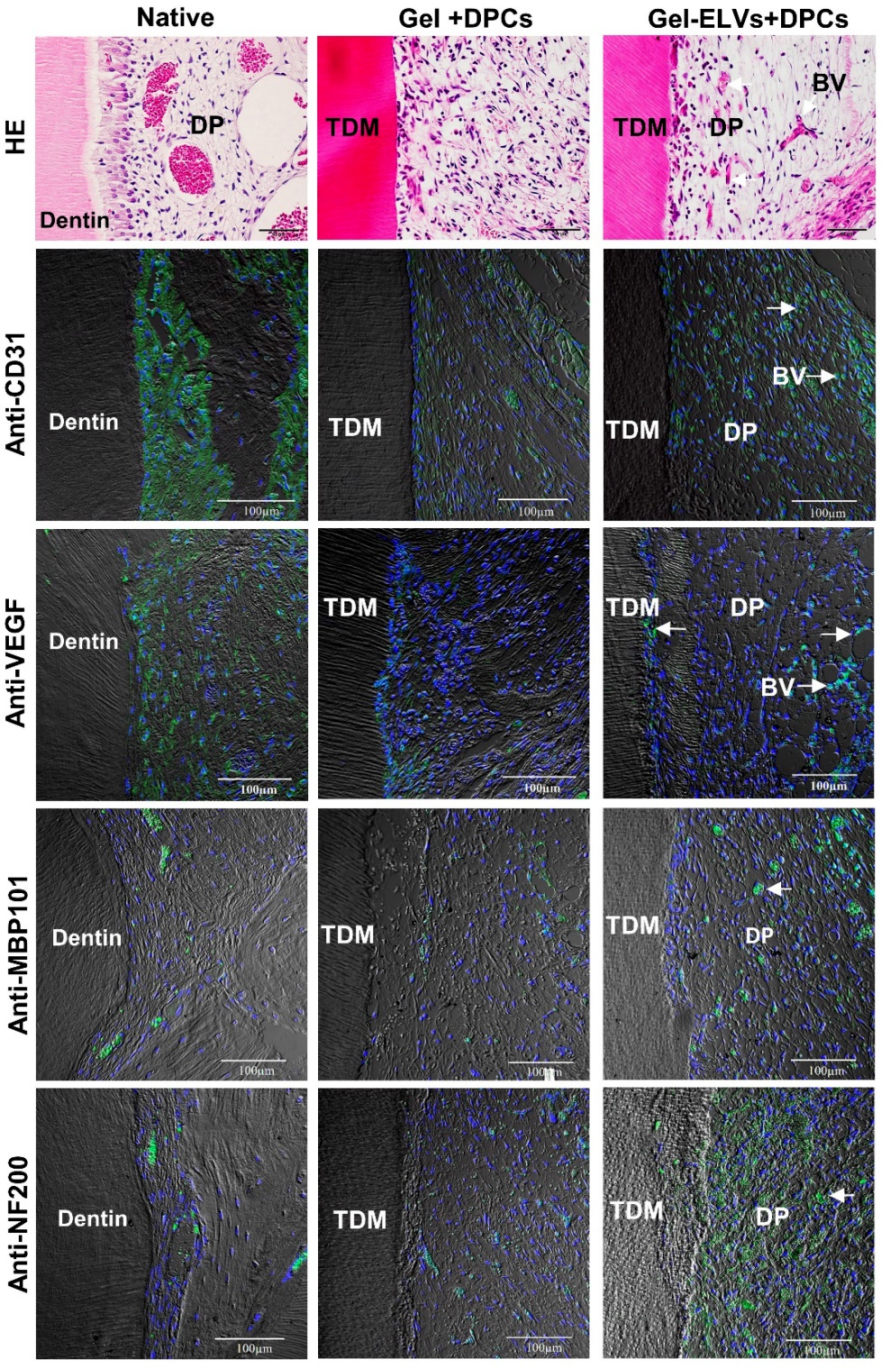
** HERS-H1 cells-derived ELVs increased angiopoiesis *in vivo*.** HE staining showing the newly-formed blood vessels (white arrows). Immunofluorescence showing the increased expression of angiogenic markers (CD31 and VEGF) and neurogenesis markers (MBP101 and NF200) in the Gel-ELVs+DPCs group (white arrows). (TDM: treated dentin matrix; BV: blood vessels; DP: dental pulp-like tissue). Scale bars are shown.

Concomitantly, H&E staining also showed that a large number of newly formed blood vessels grew into the pulp chambers of TDM (Figure 8). Immunofluorescence experiments demonstrated that sections were stained with angiogenic markers, with an increased expression of CD31 and VEGF being detected in the Gel-ELVs+DPCs group (Figure 8, Figure S2D). In addition, MBP101 and NF200 were also shown to be positively expressed in the Gel-ELVs+DPCs group (Figure 8, Figure S2D), whereas a lower expression of these neurogenic markers was demonstrated in the control. These results indicated that ELVs-H1-incorporated collagen gel could enhance the vascularization and neuralization of the implants.

## Discussion

Conventional endodontic treatments targeting irreversible pulp disease have been using biomaterials to fill the roots, however without succeeding in the regeneration of dentin or pulp tissue. Recent advances in stem cell therapy and tissue engineering have indicated that dental mesenchymal stem cells combined with biocompatible scaffolds or stem cell aggregates might have great potential in the functional regeneration of the dental pulp. However, so far only embryonic-derived dental mesenchymal stem cells have shown the powerful capacity of regeneration of the dental pulp. Moreover, the long-term *in vitro* expansion of these stem cells has also led to a senescence phenotype, with compromised differentiation potential, thus resulting in failed attempts for regeneration of the dental pulp. It was confirmed that the regenerative effects of dental stem cell-based therapy were mainly dependent on the microenvironment and the interactions between dental epithelial and dental mesenchymal cells 35, 46, 47. The formation of a functional root has been reported to depend on complex epithelial-mesenchymal interactions and the integration of the root with the blood supply and the innervations of nerves. During the development of the root of the tooth, dental papilla cells (DPCs) have been shown to interact with the Hertwig's epithelial root sheath, subsequently undergoing differentiation into odontoblasts and dental pulp cells 19. Previous studies have also demonstrated that Hertwig's epithelial root sheath cells or epithelial cell rests of Malassez are important sources of stem cells, which might be used in regenerative therapies for a variety of tooth tissues, such as dental pulp and periodontal tissues 44, 48, 49. However, HERS is known to be a fleeting, transient structure assembled in the early period of the formation and elongation of the root, making it difficult to obtain enough primary cells for biological and preclinical studies. To address this issue, our lab previously established an immortalized Hertwig's epithelial root sheath cell line named HERS-H1, which was shown to retain the characteristics of primary HERS cells in inducing the odontoblastic differentiation of dental mesenchymal cells. In the present study, we isolated exosome-like vesicles from HERS-H1 cells and found they could mediate at least in part the paracrine effects of the cell interactions of HERS cells. Accordingly, HERS cell-generated exosome-like vesicles were shown to be efficiently transported to dental mesenchymal cells, where they mimicked the microenvironment of the epithelia-mesenchyme interaction during the development of the tooth and regulated the proliferation and differentiation of dental mesenchymal stem cells. The present study was the first to report that HERS cell-derived extracellular vesicles exhibited an inductive effect in enhancing the *in vitro* proliferation and differentiation of DPC cells and contributed to the functional regeneration of the pulp with neovascularization and neuralization *in vivo*.

Tooth organogenesis requires the dental epithelial-mesenchymal interaction and exosomes have been intensely studied in these cell communications 50. Recently, exosomes from a vast variety of cell sources have been investigated for therapeutic applications. Regarding the regeneration of teeth, exosomes have been used in the regeneration of the dental pulp, periodontal ligament, dentin, and alveolar bone in animal models 32, 51. However, most of these research studies focused on exosomes derived from mesenchymal stem cells. As such, there have been no previous studies on the effects of exosomes derived from dental epithelial cells in the regeneration of dental tissues. As mentioned, paracrine or direct interaction between HERS and dental papilla cells has been reported to play an important role in the formation of the pulp-dentin tissue 19. A previous study on the development of teeth also indicated that exosomes were present in the dental epithelium and mesenchyme, and mediated the epithelial-mesenchymal interaction and functions of dental cells 31. Thus, we hypothesized that HERS-derived exosome-like vesicles might play an important role in the dental epithelial-mesenchymal interaction and cast our focus on the application of ELVs-H1 on the regeneration of pulp-dentin tissues through the promotion of the epithelial-mesenchymal interaction.

Exosomes have been demonstrated to modulate the proliferation, migration, and differentiation of target cells by transferring miRNAs, mRNAs, and proteins 52-54. In this study, our results indicated that ELVs-H1 at a concentration of 80 μg/mL could mediate the dental epithelial-mesenchymal interaction and promote the proliferation, migration, and odontoblast differentiation of DPCs. However, higher concentrations of ELVs-H1 showed a reverse effect and the mechanism of this phenomenon remained elusive. A possible explanation might be that DPCs were overloaded with proteins or nucleic acids from ELVs-H1 55. Our data also indicated that ELVs-H1 could induce odontoblastic differentiation and osteodentin formation under renal capsule. However, the regeneration of functional pulp-dentin tissue required complex structures of both hard and soft tissue. In this process, not only the odontoblast differentiation and formation of reparative dentin are known to be important, but also the regeneration of blood vessels and nerves. Exosomes were reported to have be used to maximize the local proangiogenic potential in regenerative dentistry 33, 56, 57. It has also been elaborated in several reviews that exosomes might play an important role in neurogenic niches, neurogenesis, and therapeutic strategies of neurological diseases 58-60. In this study, ELVs-H1 were shown to promote the tube-like formation of HUVECs and also led to the increased expression of neurogenic markers of DPC cells *in vitro*. From these results, it could be inferred that ELVs-H1 might at least partially substitute their parent cells in inducing the differentiation of mesenchymal cells and synthesis of matrix. These HERS-derived extracellular vesicles were demonstrated to act as a mediator in the interaction between dental epithelial and mesenchymal cells, and could be delivered into target mesenchymal stem cells thus giving rise to odontogenic, angiogenic, and ultimately neurogenic differentiation, which would be necessary for the functional regeneration of the pulp-dentin tissue.

To comprehensively understand the functional roles of ELVs-H1 on the dental pulp regeneration, a total 204 proteins were identified in the ELVs-H1 by label-free LC-MS/MS quantitation technologies. GO-based category clustering and KEGG pathway mapping indicated that ELVs-H1 may impact cell migration, interaction between cells and their local extracellular matrix and PI3K-Akt signaling pathway, which were previously reported involving the dental pulp development and regeneration^61^-^64^. The Wnt/β-catenin signaling between the dental epithelium and mesenchyme has been reported to play multiple roles in the morphogenesis of teeth 19, 43, 65. Studies have also implied that the Wnt/β-catenin signaling might be required in odontoblasts during the development of the root of the tooth 44. Research has indicated that Wnt proteins could be secreted on extracellular vesicles with this fraction representing an active form of secreted Wnt proteins 66. In the current study, we found that ELVs-H1 contained Wnt3a proteins and treatment with ELVs was reported to significantly upregulate the expression of β-catenin and Wnt/β-catenin target genes, also leading to the translocation of β-catenin into the nucleus *in vitro*, with this effect being obviously attenuated by treatment with DKK1, indicating the ELVs-H1 activated the Wnt/β-catenin signaling pathway in DPC cells. Further *in vivo* results also confirmed that ELVs-H1 could promote the activation of the Wnt/β-catenin pathway. Generally, Wnt signaling is known to initiate when a Wnt protein binds to the N-terminal extra-cellular cysteine-rich domain of a Frizzled (Fz) family receptor 67. Therefore, we suspected that Wnt proteins, such as Wnt3a could be secreted on ELVs-H1 and then bind to the Wnt receptor, promoting the activation of downstream signals. A previous study has also indicated that the Wnt/β-catenin signaling played an important role in the angiogenesis, odontoblastic differentiation, and neural differentiation of dental mesenchymal stem cells, and acted as an important signal in enhancing the regeneration of the pulp-dentin tissue 68-71. Therefore, from a perspective of signaling pathways, our study demonstrated that HERS-derived ELVs could induce the differentiation of dental mesenchyme cells, in the absence of epithelium cells, potentially by activating pivotal signaling pathways, such as Wnt/β-catenin, thus providing the possibility for the restoration of the functional pulp-dentin tissue.

In order to use ELVs-H1 as therapeutic agents locally, these vesicles should be compatible for use with any materials that might be applied in applications of regenerative medicine. One way of estimating this ability is to evaluate the binding and releasing of ELVs-H1 to structural ECM proteins. Previously, various biomaterials have been used for exosome-mediated tissue regeneration, such as collagen, matrigel, chitosan, and hydrogel 32, among which collagen has been one of the most common materials. The collagen gel used in our study is known as a kind of biological material characterized by its high biocompatibility, and has been widely used as a carrier for delivering dental cells or substrates in applications of regeneration of tooth tissues 5, 47, 74, 75. Basic levels of ELVs-H1 were shown to result in the accelerated proliferation and differentiation of DPCs. However, the sustained release of ELVs-H1 was demonstrated to be clearly more effective than free ELVs-H1. Our results indicated that ELVs-H1 could bind to the collagen gel and the sustained release test performed demonstrated that ELVs-H1 were gradually released from the collagen gel. More importantly, the continuously-released ELVs-H1 were reported to be efficiently endocytosed by DPC cells. Of note, a sustained release system is desirable for prolonging the retention of bioactive ELVs. In our study, this highly biocompatible ELVs-H1-collagen provided dental mesenchymal stem cells with a suitable microenvironment for odontoblast differentiation and opened up an avenue of research in the combined use of ELVs-H1 and biomaterials in the regeneration of dental pulp-dentin tissues. In this study, we further investigated the use of ELVs-H1-incorporated collagen gel to generate engineered pulp-dentin tissue in the tooth root slice model. Results indicated that ELVs-H1 triggered the regeneration of dental pulp-like tissue. We were encouraged to find an increased expression of DSPP and DMP1 at the interface of the hard and soft tissue, indicating enhanced odontogenic differentiation 4, 76. The formation of reparative-dentin-like tissue is considered to be a key process for the functional regeneration of the pulp-dentin tissue and the fact that ELVs-H1 triggered this process was explicitly indicative of its potential in pulp-dentin regenerative therapy. In addition, our results also showed that ELVs-H1 triggered a more robust vascularization and a higher expression of the angiogenic and neurogenic marker proteins. Taken together, these results indicated the potential of ELVs-H1 as tools in regenerating vascularized pulp tissue accompanied with reparative-dentin-like structures and neuroid tissues.

The trophic effect is known to be required in guaranteeing the effective coordination among different cell types within tissues. The most classical means of this effect has been reported to be represented by signaling through secreted and soluble factors, which connect neighboring cells acting in a paracrine or even endocrine manner, In our study, the trophic effect of HERS supported the regeneration of the pulp-dentin tissue by delivering ELVs-H1 containing bioactive molecules, such as Wnt proteins enhancing the activity of tissue-resident cells. However, as ELVs-H1 might be likely to carry a cargo of multiple molecules collectively acting on effector cells, we hypothesized that the ELVs-mediated changes observed in our study might have been the result of the function of both endocytic proteins and miRNA. This study only explored the role of Wnt/β-catenin signaling in inducing the differentiation of DPCs cells. Further studies are required to characterize more mechanisms by which ELVs-H1 might be able to control the fate of dental mesenchymal stem cells.

## Conclusion

In this study, we used ELVs derived from immortalized HERS-H1 cells to trigger the regeneration of pulp-dentin tissue and confirm the possibility of using ELVs-H1 to generate engineered dental pulp. The ELVs-H1 provided a proper microenvironment to mimic the epithelia-mesenchyme interactions during the development of the tooth and regulated the differentiation of dental mesenchymal stem cells by activating the Wnt/β-catenin pathway. We hypothesized that along with the incorporation of extracellular matrix biomaterials, ELVs could be used to recreate a complete extracellular microenvironment that would enable the safe and reliable differentiation of dental mesenchymal stem cells. We believe that our results might serve as a starting point to further investigate the possibility of using epithelium-derived ELVs in mediating interactions between dental epithelial and mesenchymal cell and regenerating functional tooth tissue.

## Supplementary Material

Supplementary figures and tables.

## Abbreviations

HERS: Hertwig's epithelial root sheath cells
DPCs: dental papilla cells
ELVs: exosome-like vesicles
ELVs-H1: exosome-like vesicles derived from Hertwig's epithelial root sheath cell line H1
CCK-8: Cell Counting Kit-8
SHH: sonic hedgehog
BMP: bone morphogenetic protein
DKK1: dickkopf WNT signaling pathway inhibitor 1
EMI: epithelial-mesenchymal interaction
EDTA: ethylenediaminetetraacetic acid
HUVECs: human umbilical vein endothelial cells
NM: neurogenic medium
TMD: treated dentin matrix
OCN: osteocalcin
DSPP: dentin sialophosphoprotein
DMP1: dentin matrix protein 1
ALP: alkaline phosphatase
RUNX2: runt related transcription factor 2
VEGF: vascular endothelial growth factor
HSP70: 70 kilodalton heat shock proteins
CNC: cranial neural crest
AXIN2: axis inhibition protein 2
TCF7: transcription factor 7
