# Supplementary material for: Exosome-like vesicles derived from Hertwig's epithelial root sheath cells promote the regeneration of dentin-pulp tissue
Source: Theranostics. 2020 Apr 27;10(13):5914–31. doi: 10.7150/thno.43156 (PMC7254987; doi:10.7150/thno.43156)
Supplement: Supplementary file 1 — Supplementary figures and tables. [file thnov10p5914s1.pdf]

Supplementary information

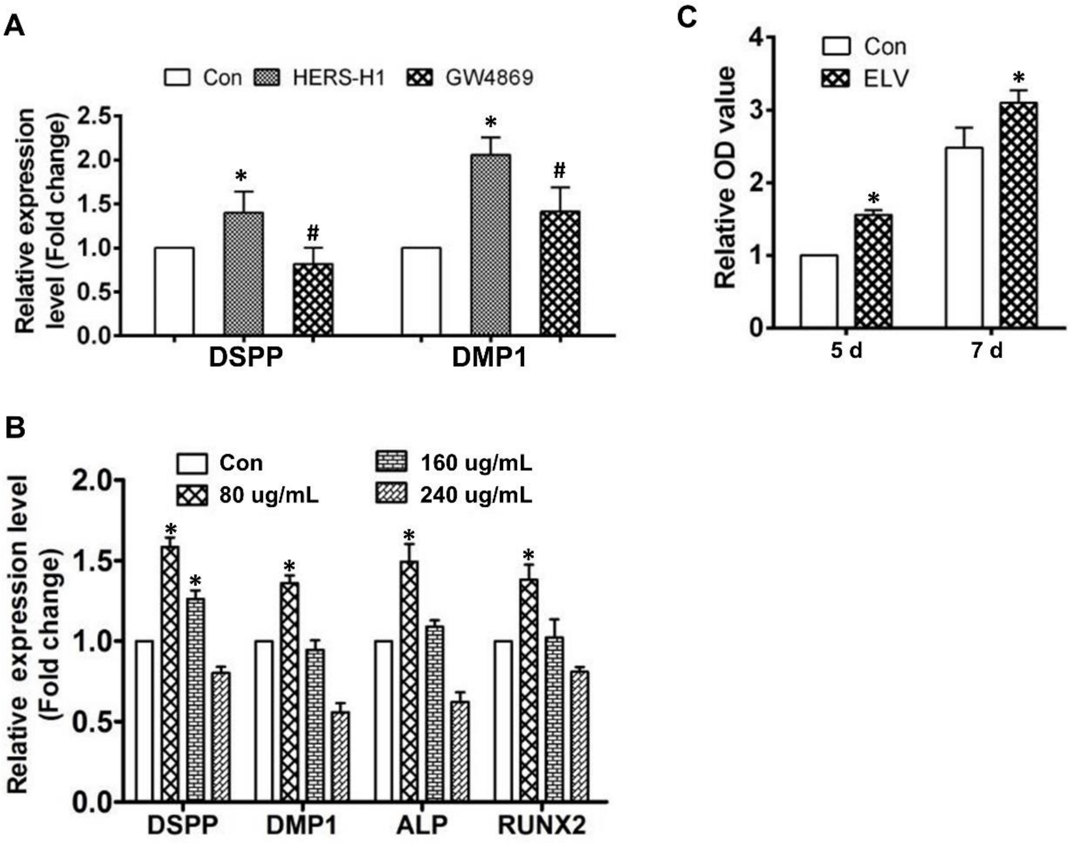

Supplementary Figure S1. (A) Quantitative analysis of western blotting showed HERS-H1 cells upregulated the expression of DSPP and DMP1 in DPCs cells, which was attenuated by pretreatment with GW4869; (B) Quantitative analysis of western blotting showed upregulated expression of odontoblastic markers (DSPP, DMP1, ALP, and RUNX2) in DPC cells after treatment with various concentrations of ELVs for 3 d; (C) The quantitative analysis of calcium mineralization showed that treatment with ELVs increased the odontogenic differentiation of DPC cells. *$p < 0.05$ vs. Con; #$p < 0.05$ vs HERS-H1.

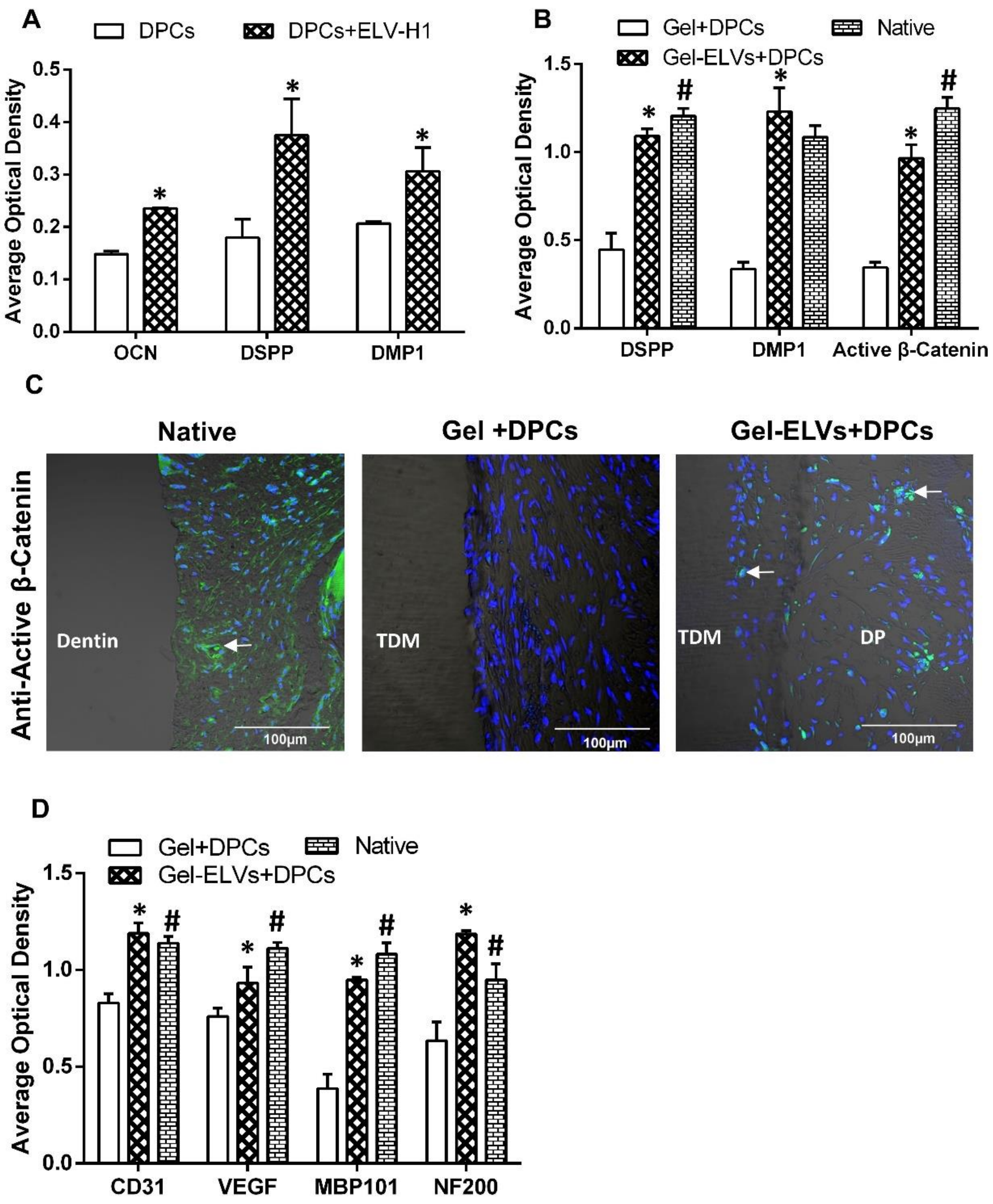

Supplementary Figure S2. (A) Quantitative analysis of the immunohistochemical staining was performed by measuring the average optical density; (B) A quantitative analysis of the immunofluorescent staining for odontoblast marker differentiation was performed by measuring the average optical density; (C) Immunofluorescence showed the expression of β-Catenin was upregulated in the Gel-ELVs+DPCs group (white arrows). (D) Quantitative analysis of the immunofluorescent staining for angiogenic markers (CD31 and VEGF) and neurogenesis markers (MBP101 and NF200) was performed by measuring the average optical density. Scar bars are shown, $*p < 0.05$ vs. Con; $\#p < 0.05$ vs HERS-H1.

Table S1 The proteins information identified by LC–MS/MS in ELVs-H1

Uniprot ID	Description	Gene
A0A096P6L8	Fibronectin	Fn1
F1LST1	Fibronectin	Fn1
P06238	Alpha-2-macroglobulin	A2m
D3ZK14	Tenascin N	Tnn
Q1PBJ1	Lactadherin	Mfge8
Q07936	Annexin A2	Anxa2
F1M077	Protein Wnt	Wnt3a
O70513	Galectin-3-binding protein	Lgals3bp
P48037	Annexin A6	Anxa6
P14668	Annexin A5	Anxa5
Q64599	Hemiferrin	LOC286987
A0A0G2K3K2	Actin, cytoplasmic 1	Actb
Q9QZK5	Serine protease HTRA1	Htra1
Q9QZA2	Programmed cell death 6-interacting protein	Pdcd6ip
B2LYI9	Tenascin C	Tnc
P63018	Heat shock cognate 71 kDa protein	Hspa8
A0A0G2K1L0	Tenascin C	Tnc
Q6P762	Alpha-mannosidase	Man2b1
Q7TPB1	T-complex protein 1 subunit delta	Cct4
A0A0G2KAJ7	Collagen alpha-1(XII) chain	Col12a1
Q9QZD1	Chemokine (C-X-C motif) ligand 12 (Stromal cell-derived factor 1)	Cxcl12
P07150	Annexin A1	Anxa1
B5DEH7	C1r protein	C1r
G3V928	Prolow-density lipoprotein receptor-related protein 1	Lrp1
F1M779	Clathrin heavy chain	Cltc
P06685	Sodium/potassium-transporting ATPase subunit alpha-1	Atp1a1
F1M790	Prostaglandin F2 receptor negative regulator	Ptgfrn
F1M9B2	Insulin-like growth factor binding protein 7, isoform CRA_b	Igfbp7
F1MAA7	Laminin subunit gamma 1	Lamc1
P55260	Annexin A4	Anxa4
C0JPT7	Filamin A	Flna
Q62812	Myosin-9	Myh9
Q5U367	Multifunctional procollagen lysine hydroxylase and glycosyltransferase LH3	Plod3
P69897	Tubulin beta-5 chain	Tubb5
Q6IRE4	Tumor susceptibility gene 101 protein	Tsg101
A0A0G2JSH5	Serum albumin	Alb
Q71SA3	Thrombospondin 1	Thbs1

Q62894	Extracellular matrix protein 1	Ecm1
Q6MG90	Complement C4B (Chido blood group)	C4b
F1LR87	Beta-hexosaminidase	Hexb
Q5XIM9	T-complex protein 1 subunit beta	Cct2
D3ZFH5	Inter-alpha-trypsin inhibitor heavy chain 2	Itih2
Q5M7T5	Serine (Or cysteine) peptidase inhibitor, clade C (Antithrombin), member 1	Serpinc1
F1MAN8	Laminin subunit alpha 5	Lama5
M0R757	Elongation factor 1-alpha	LOC100360413
G3V763	Collagen alpha-1(V) chain	Col5a1
D4A8G5	Transforming growth factor, beta-induced	Tgfbi
G3V852	RCG55135, isoform CRA_b	Tln1
Q9ERB4	Versican core protein (Fragments)	Vcan
D4AC23	T-complex protein 1 subunit eta	Cct7
Q641X3	Beta-hexosaminidase subunit alpha	Hexa
P68370	Tubulin alpha-1A chain	Tuba1a
P62804	Histone H4	H4c2
Q9JI92	Syntenin-1	Sdcbp
A0A4X0WLY2	Matrix extracellular phosphoglycoprotein	Mepe
F7EPE0	Prosaposin	Psap
G3V8H7	Olfactomedin-like 3 (Predicted)	Olfml3
P20909	Collagen alpha-1(XI) chain	Col11a1
Q6P502	T-complex protein 1 subunit gamma	Cct3
Q10758	Keratin, type II cytoskeletal 8	Krt8
A0A0G2K2V6	Keratin, type I cytoskeletal 10	Krt10
P00762	Anionic trypsin-1	Prss1
A0A0G2JXI9	Histone H2B	Hist1h2bo
Q4FZT9	26S proteasome non-ATPase regulatory subunit 2	Psmd2
Q68FP1	Gelsolin	Gsn
A0A140TAE0	Major vault protein	Mvp
M0R6K0	Laminin subunit beta-2	Lamb2
P40329	Arginine--tRNA ligase, cytoplasmic	Rars
P01830	Thy-1 membrane glycoprotein	Thy1
Q4FZU2	Keratin, type II cytoskeletal 6A	Krt6a
Q5RJN2	Pcolce protein	Pcolce
D3ZVK7	Histone H2A	Hist1h2ak
A0A0G2JV65	14-3-3 protein zeta/delta	Ywhaz
P55063	Heat shock 70 kDa protein 1-like	Hspa1l
A0A0G2K926	Murinoglobulin-1	LOC297568
M0R660	Glyceraldehyde-3-phosphate dehydrogenase	
D4ACB8	Chaperonin subunit 8 (Theta) (Predicted), isoform CRA_a	Cct8
F1M6Q3	Collagen type IV alpha 2 chain	Col4a2

D3ZAF5	Periostin	Postn
D3ZQN7	Laminin subunit beta 1	Lamb1
P06761	Endoplasmic reticulum chaperone BiP	Hspa5
A0A0H2UHQ0	4F2 cell-surface antigen heavy chain	Slc3a2
D3ZQM3	Integrin alpha 3 variant B	Itga3
M0RBF1	Complement C3	C3
P12785	Fatty acid synthase	Fasn
Q68FQ0	T-complex protein 1 subunit epsilon	Cct5
Q68EJ2	A disintegrin and metalloproteinase with thrombospondin motifs 1	Adamts1
A0A0G2JST3	Keratin, type II cytoskeletal 1	Krt1
P05197	Elongation factor 2	Eef2
P31977	Ezrin	Ezr
P34058	Heat shock protein HSP 90-beta	Hsp90ab1
A0A096MK30	Moesin	Msn
P28480	T-complex protein 1 subunit alpha	Tcp1
M0R9X8	Cytoplasmic dynein 1 heavy chain 1	Dync1h1
Q6IG02	Keratin, type II cytoskeletal 2 epidermal	Krt2
Q80ZA3	Alpha-2 antiplasmin	Serpinf1
P04642	L-lactate dehydrogenase A chain	Ldha
D3ZE04	Collagen type VII alpha 1 chain	Col7a1
Q3MID7	Lipopolysaccharide-binding protein	Lbp
F7FHT4	Semaphorin 3C	Sema3c
P00787	Cathepsin B	Ctsb
A0A0G2JZH3	Thrombospondin 3	Thbs3
D3ZUL3	Collagen type VI alpha 1 chain	Col6a1
F1MA59	Collagen type IV alpha 1 chain	Col4a1
G3V6B1	Transforming growth factor beta	Tgfb2
Q9EPB1	Dipeptidyl peptidase 2	Dpp7
Q5XI77	Annexin	Anxa11
Q8R3Z7	EH-domain-containing 4	Ehd4
A0A0G2JWB6	Peroxidasin	Pxdn
Q4V8N0	Lipocalin 7, isoform CRA_a	Tinagl1
G3V843	Prothrombin	F2
Q5BJU0	RAS-related 2	Rras2
P46462	Transitional endoplasmic reticulum ATPase	Vcp
F1LQ00	Collagen type V alpha 2 chain	Col5a2
A0A0G2JZI2	Glutamyl-prolyl-tRNA synthetase	Eprs
A0A0G2K0M8	Neural cell adhesion molecule 1	Ncam1
Q6IRJ7	Annexin	Anxa7
D3ZBS2	Inter-alpha-trypsin inhibitor heavy chain H3	Itih3
G3V824	Insulin-like growth factor 2 receptor	Igf2r
Q6P9V1	Tetraspanin	Cd81
P53987	Monocarboxylate transporter 1	Slc16a1

A0A0G2JSW3	Globin a4	Hbb
Q05BA4	Myadm protein	Myadm
P49134	Integrin beta-1	Itgb1
O70352	CD82 antigen	Cd82
O08591	Perlecan (Fragment)	Hspg2
Q6LDG8	Alpha-2-macroglobulin (Fragment)	
P16617	Phosphoglycerate kinase 1	Pgk1
P62982	Ubiquitin-40S ribosomal protein S27a	Rps27a
G3V8C3	Vimentin	Vim
G3V633	FMS-like tyrosine kinase 1, isoform CRA_a	Flt1
P62909	40S ribosomal protein S3	Rps3
Q63803	Guanine nucleotide-binding protein G(s) subunit alpha isoforms XLas	Gnas
P08289	Alkaline phosphatase, tissue-nonspecific isozyme	Alpl
Q63772	Growth arrest-specific protein 6	Gas6
B5DFK1	Coatomer subunit alpha	Copa
E9PU28	Inosine-5-monophosphate dehydrogenase 2	Impdh2
Q32KJ5	N-acetylglucosamine-6-sulfatase	Gns
Q63016	Large neutral amino acids transporter small subunit 1	Slc7a5
A0A0G2K9Q9	Keratin, type I cytoskeletal 17	Krt17
P42123	L-lactate dehydrogenase B chain	Ldhb
A0A0G2K8V2	Vinculin	Vcl
P62260	14-3-3 protein epsilon	Ywhae
Q5PPG2	Legumain	Lgmn
A0A0H2UHA1	Mannan-binding lectin serine protease 1	Masp1
P05065	Fructose-bisphosphate aldolase A	Aldoa
P60901	Proteasome subunit alpha type-6	Psma6
O88753	Epsilon 2 globin	Hbe2
B2RYM3	Inter-alpha trypsin inhibitor, heavy chain 1	Itih1
Q66HT5	CCN family member 1	Ccn1
Q6IFU7	Keratin, type I cytoskeletal 42	Krt42
G3V7Z4	Glia-derived nexin	Serpine2
A0A0H2UHS7	60S ribosomal protein L18	Rpl18
A0A0G2K2B8	14-3-3 protein eta	Ywhah
P40241	CD9 antigen	Cd9
A0A0G2K9H8	ADP-ribosylation factor 3	Arf3
Q4KLZ0	Vanin 1	Vnn1
D3ZBN3	Eph receptor A2	Epha2
Q4QQW8	Putative phospholipase B-like 2	Plbd2
P28648	CD63 antigen	Cd63
P12928	Pyruvate kinase PKLR	Pklr
Q6IRK9	Carboxypeptidase Q	Cpq

B5DFC8	Eukaryotic translation initiation factor 3 subunit C	Eif3c
Q5U2S7	Proteasome (Prosome, macropain) 26S subunit, non-ATPase, 3	Psmd3
P62836	Ras-related protein Rap-1A	Rap1a
A0A140TAA1	Immunoglobulin superfamily, member 8	Igsf8
B2GVB9	Fermitin family member 3	Fermt3
A0A0G2K2R5	EGF-containing fibulin extracellular matrix protein 2	Efemp2
F1MAD9	Structural maintenance of chromosomes protein	Smc4
P14562	Lysosome-associated membrane glycoprotein 1	Lamp1
A0A0G2K6Q8	Protein Wnt	Wnt10b
Q9JMJ4	Pre-mRNA-processing factor 19	Prpf19
M0RAR2	ITI_HC_C domain-containing protein	
A0A0H2UHX3	40S ribosomal protein S4	Rps4x-ps9
G3V7G9	Eukaryotic translation initiation factor 3, subunit 6 interacting protein	Eif3l
D3ZAT4	Serine (Or cysteine) peptidase inhibitor, clade A (Alpha-1 antiproteinase, antitrypsin), member 9 (Predicted)	Serpina9
P13941	Collagen alpha-1(III) chain	Col3a1
D3ZUY8	AP-2 complex subunit alpha	Ap2a1
D3ZQ25	Fibulin-1	Fbln1
D4A8H8	Cytoplasmic FMR1-interacting protein	Cyfip1
A0A0G2JSV6	Globin c2	Hba-a2
G3V888	ATP-citrate synthase	Acly
A0A0G2JUA5	AHNAK nucleoprotein	Ahnak
Q7TMA5	Apolipoprotein B-100	Apob
Q1JU68	Eukaryotic translation initiation factor 3 subunit A	Eif3a
P18420	Proteasome subunit alpha type-1	Psma1
P63164	Small nuclear ribonucleoprotein-associated protein N	Snrpn
Q66X93	Staphylococcal nuclease domain-containing protein 1	Snd1
A0A0G2K896	Similar to RIKEN cDNA 1300017J02	RGD1310507
P08934	Kininogen-1	Kng1
Q63041	Alpha-1-macroglobulin	A1m
G3V6X1	Fibulin 2	Fbln2
P49744	Thrombospondin-4	Thbs4
Q71DI1	Dermcidin	
A0A0G2K1Y8	Spectrin alpha chain, non-erythrocytic 1	Sptan1

G3V722	Beta-1,4-galactosyltransferase 1	B4galt1
Q6AYS3	Carboxypeptidase	Ctsa
D3ZP82	Lysyl oxidase-like 3	Loxl3
A0A0G2K9I5	Similar to H3 histone, family 3B	RGD1564548
O88775	Embigin	Emb
A0A0G2K7G7	Septin 8	Sept8
F1LTF8	Laminin subunit alpha 4	Lama4
F1LND0	Collagen type XVI alpha 1 chain	Col16a1
A0A0G2K151	Apolipoprotein E	Apoe

Table S2 GO annotation of biological process of proteins in ELVs-H1

Term	P Value	Genes
GO:0007155~cell adhesion	3.26E-19	TLN1, IGFBP7, TNC, POSTN, ITGB1, VCL, CD9, LGALS3BP, LAMB2, TGFBI, COL12A1, EMB, THBS1, THBS3, THBS4, FN1, B4GALT1, MFGE8, ITGA3, MYH9, COL16A1, TINAGL1, COL5A1, NCAM1, LAMA4, LAMA5, VCAN, LAMC1
GO:1904871~positive regulation of protein localization to Cajal body	3.62E-11	CCT7, TCP1, CCT5, CCT4, CCT8, CCT2, CCT3
GO:0098609~cell-cell adhesion	9.11E-10	HSP90AB1, LDHA, YWHAZ, EEF2, YWHAE, CXCL12, EPHA2, ANXA2, RARS, SND1, CCT8, FASN, SDCBP, HSPA5, AHNAK, SPTAN1, EHD4
GO:0007339~binding of sperm to zona pellucida	3.24E-09	HSPA1L, CCT7, B4GALT1, TCP1, CCT5, CCT4, CCT8, CCT2, CCT3
GO:1904874~positive regulation of telomerase RNA localization to Cajal body	6.07E-09	CCT7, TCP1, CCT5, CCT4, CCT8, CCT2, CCT3
GO:0070527~platelet aggregation	9.76E-09	ACTB, TLN1, FERMT3, WNT3A, GNAS, MYH9, HBB, FLNA, VCL
GO:1904851~positive regulation of establishment of protein localization to telomere	1.55E-08	CCT7, TCP1, CCT5, CCT4, CCT8, CCT2
GO:0010951~negative regulation of endopeptidase activity	5.91E-08	KNG1, A2M, LOC297568, C4B, C3, SERPINA9, LRP1, SERPINE2, SERPINF1, ITIH1, SERPINC1, ITIH2, ITIH3
GO:0009611~response to wounding	1.70E-07	B4GALT1, A2M, SERPINE2, TNC, CD81, F2, VCAN, CTSB, TGFB2, FN1
GO:1901998~toxin transport	1.87E-07	CCT7, TCP1, CCT5, CCT4, CCT8, CCT2, HSPA5, CCT3
GO:0035987~endodermal cell differentiation	4.08E-07	COL4A2, COL7A1, COL12A1, COL6A1, LAMB1, COL11A1, FN1
GO:0032212~positive regulation of telomere maintenance via telomerase	1.15E-06	CCT7, TCP1, CCT5, CCT4, CCT8, CCT2, CCT3
GO:0030335~positive regulation of cell migration	1.99E-06	FLT1, FERMT3, RRAS2, SEMA3C, SDCBP, HSPA5, LAMB1, THBS1, ITGB1, MYADM, CXCL12, FN1, TGFB2
GO:0030199~collagen fibril organization	3.74E-06	PLOD3, COL3A1, COL5A2, COL11A1, COL5A1, ANXA2, TGFB2
GO:0001649~osteoblast differentiation	4.97E-06	ALPL, SND1, TNC, WNT3A, RRAS2, FASN, COL6A1, VCAN, CLTC, EPHA2
GO:0016477~cell migration	8.42E-06	FLT1, LAMA5, TNN, LAMC1, LAMB1, CD63, THBS1, CXCL12, ITGB1, EPHA2, COL5A1, TGFB2

GO:0030198~extracellular matrix organization	8.91E-06	B4GALT1, FBLN1, PXDN, WNT3A, TGFBI, HSPG2, POSTN, TGFB2, FN1
GO:0050821~protein stabilization	1.02E-05	CCT7, LAMP1, TCP1, CCT5, WNT10B, CCT4, CCT8, CCT2, CCT3, FLNA
GO:0001501~skeletal system development	1.39E-05	HEXA, HEXB, COL3A1, VCAN, GNAS, MEPE, COL5A2, EPHA2, TGFB2
GO:0030855~epithelial cell differentiation	1.89E-05	ANXA7, COL4A1, EZR, KRT10, CTSB, PGK1, CD63, ANXA4
GO:0008360~regulation of cell shape	5.96E-05	ALDOA, ANXA7, EZR, HEXB, F2, ANXA1, MSN, MYH9, FN1
GO:0050766~positive regulation of phagocytosis	1.10E-04	C3, RAP1A, MFGE8, MYH9, HSPA8, GAS6
GO:0006457~protein folding	1.91E-04	CCT7, HSP90AB1, TCP1, CCT5, CCT4, CCT8, CCT3, HSPA8
GO:0051603~proteolysis involved in cellular protein catabolic process	2.01E-04	PSMA6, LGMN, CTSA, HSPA5, CTSB, TINAGL1
GO:0009314~response to radiation	2.21E-04	SERPINE2, VIM, COL3A1, HSPA5, CXCL12, TGFB2
GO:0030195~negative regulation of blood coagulation	2.41E-04	KNG1, SERPINE2, APOE, ANXA5
GO:0007229~integrin-mediated signaling pathway	3.26E-04	FBLN1, LAMA5, FERMT3, COL3A1, ITGA3, ADAMTS1, ITGB1
GO:0005975~carbohydrate metabolic process	3.76E-04	B4GALT1, LDHB, LDHA, HEXA, HEXB, PKLR, SLC3A2, PGK1
GO:0006956~complement activation	4.86E-04	MASP1, C3, C4B, C1R
GO:0061028~establishment of endothelial barrier	5.94E-04	EZR, RAP1A, MSN, MYADM
GO:0010628~positive regulation of gene expression	6.25E-04	FBLN1, APOB, EZR, GSN, TNC, VIM, ITGA3, MSN, HSPA8, GAS6, RPS3, FN1, TGFB2
GO:0090666~scaRNA localization to Cajal body	6.62E-04	TCP1, CCT4, CCT2
GO:0006953~acute-phase response	6.83E-04	KNG1, A2M, F2, LBP, FN1
GO:0001525~angiogenesis	7.88E-04	FLT1, TGFBI, MFGE8, MYH9, ECM1, FLNA, ANXA2, THY1, FN1
GO:0009612~response to mechanical stimulus	8.44E-04	ACTB, TNC, COL3A1, POSTN, CTSB, THBS1, CXCL12
GO:0048146~positive regulation of fibroblast proliferation	8.82E-04	KNG1, FBLN1, FLT1, GAS6, ANXA2, FN1
GO:0007162~negative regulation of cell adhesion	9.13E-04	KNG1, FBLN1, TNC, HSPG2, LAMB1
GO:0034446~substrate adhesion-dependent cell spreading	9.13E-04	LAMA5, FERMT3, LAMC1, LAMB1, FN1
GO:0031623~receptor internalization	0.0010013	CD9, EZR, CD81, CLTC, ITGB1
GO:2000427~positive regulation of	0.0010959	C3, C4B, MFGE8

apoptotic cell clearance		
GO:0045109~intermediate filament organization	0.0011721	KRT17, VIM, KRT2, KRT10
GO:0032355~response to estradiol	0.0013917	APOB, FLT1, C3, VIM, ANXA1, EEF2, POSTN, HSPA8, TGFB2
GO:0042493~response to drug	0.0015426	HSP90AB1, LDHA, YWHAZ, HSPG2, ANXA1, EEF2, ATP1A1, ITGA3, ITGB1, TGFB2, NCAM1, GNAS, THBS1, HSPA8, FN1
GO:0061684~chaperone-mediated autophagy	0.0016323	HSP90AB1, CTSA, HSPA8
GO:0021762~substantia nigra development	0.0016568	ACTB, LDHA, YWHAH, HSPA5, YWHAE
GO:0007568~aging	0.0020105	NCAM1, FLT1, LRP1, SERPINF1, APOE, GSN, VIM, COL3A1, EEF2, HSPA8, TGFB2
GO:0042060~wound healing	0.0022617	B4GALT1, GSN, TNC, COL3A1, POSTN, TGFB2, FN1
GO:0033622~integrin activation	0.0022693	FERMT3, COL16A1, FN1
GO:0007159~leukocyte cell-cell adhesion	0.0022745	EZR, FERMT3, MSN, ITGB1
GO:0042311~vasodilation	0.0022745	KNG1, APOE, ALB, PLOD3
GO:0007566~embryo implantation	0.002393	FBLN1, SERPINE2, IGFBP7, HSPG2, LAMB1
GO:0031532~actin cytoskeleton reorganization	0.0025633	EZR, ANXA1, MYH9, FLNA, SPTAN1
GO:0071353~cellular response to interleukin-4	0.0028479	HSP90AB1, FASN, HSPA5, IMPDH2
GO:0043588~skin development	0.0029286	COL3A1, ITGA3, GNAS, COL5A2, COL5A1
GO:0010976~positive regulation of neuron projection development	0.0038163	SERPINE2, SERPINF1, RAP1A, ITGA3, ADAMTS1, HSPA5, ITGB1
GO:0006898~receptor-mediated endocytosis	0.0039005	LGALS3BP, LRP1, APOE, LOXL3, TINAGL1, CLTC
GO:0090004~positive regulation of establishment of protein localization to plasma membrane	0.0042428	EZR, ITGA3, ITGB1, EPHA2
GO:0006954~inflammatory response	0.0045223	KNG1, LOC297568, C3, C4B, ANXA1, VNN1, THBS1, CXCL12, ECM1, EPHA2
GO:0006508~proteolysis	0.0046177	MASP1, CPQ, LGMN, PRSS1, C1R, CTSA, TINAGL1, PCOLCE, HTRA1, F2, ADAMTS1, CTSB, DPP7
GO:0010952~positive regulation of peptidase activity	0.004762	FBLN1, PCOLCE, FN1
GO:0016525~negative regulation of angiogenesis	0.0052949	COL4A2, SERPINF1, THBS1, EPHA2, THBS4
GO:0007596~blood coagulation	0.0052949	SERPINE2, C3, F2, ANXA5, GAS6
GO:0048514~blood vessel	0.0055185	FLT1, COL4A1, THBS1, EPHA2

morphogenesis		
GO:0018149~peptide cross-linking	0.0059893	COL3A1, ANXA1, THBS1, FN1
GO:0001568~blood vessel development	0.0065196	LAMA4, COL3A1, EPHA2, COL5A1, TGFB2
GO:0001938~positive regulation of endothelial cell proliferation	0.0065196	KNG1, HSPG2, CXCL12, ECM1, THBS4
GO:0071404~cellular response to low-density lipoprotein particle stimulus	0.0068876	CD9, CD81, ITGB1
GO:0014012~peripheral nervous system axon regeneration	0.0068876	NCAM1, APOE, TNC
GO:0008347~glial cell migration	0.0068876	VCAN, TGFB2, FN1
GO:0008284~positive regulation of cell proliferation	0.0075382	WNT10B, FLT1, WNT3A, TNC, MFGE8, CXCL12, ITGB1, TGFB2, F2, CD81, SDCBP, GNAS, FN1
GO:0030212~hyaluronan metabolic process	0.0080834	ITIH1, ITIH2, ITIH3
GO:0071230~cellular response to amino acid stimulus	0.0082972	COL4A1, COL3A1, COL6A1, COL16A1, COL5A2
GO:0030334~regulation of cell migration	0.0086871	LAMA4, LAMA5, CXCL12, FLNA, VCL
GO:0007160~cell-matrix adhesion	0.0086871	COL3A1, TNN, CD63, ITGB1, FN1
GO:0009408~response to heat	0.0095024	ALDOA, IGFBP7, PKLR, CXCL12, HSPA8

Table S3 Pathway mapping of proteins in ELVs-H1

Term	P Value	Genes
rno04512:ECM-receptor interaction	5.72E-17	COL4A2, COL4A1, TNC, COL3A1, ITGA3, ITGB1, COL5A2, COL5A1, LAMA4, LAMB2, LAMA5, COL6A1, TNN, LAMC1, LAMB1, THBS1, COL11A1, THBS3, THBS4, FN1
rno04510:Focal adhesion	1.08E-15	TLN1, TNC, COL3A1, ITGB1, VCL, LAMB2, COL6A1, TNN, LAMB1, THBS1, COL11A1, THBS3, THBS4, FN1, ACTB, COL4A2, FLT1, COL4A1, ITGA3, COL5A2, COL5A1, FLNA, LAMA4, LAMA5, RAP1A, LAMC1
rno04151:PI3K-Akt signaling pathway	6.07E-11	HSP90AB1, YWHAZ, TNC, COL3A1, ITGB1, LAMB2, COL6A1, TNN, LAMB1, THBS1, COL11A1, THBS3, THBS4, FN1, COL4A2, FLT1, COL4A1, ITGA3, COL5A2, YWHAE, EPHA2, COL5A1, LAMA4, YWHAH, LAMA5, LAMC1
rno05146:Amoebiasis	1.77E-09	COL4A2, COL4A1, COL3A1, COL5A2, COL5A1, TGFB2, VCL, LAMA4, LAMB2, LAMA5, GNAS, LAMC1, LAMB1, COL11A1, FN1
rno04974:Protein digestion and absorption	1.21E-07	COL4A2, COL4A1, COL7A1, COL3A1, SLC3A2, PRSS1, COL12A1, COL6A1, ATP1A1, COL5A2, COL11A1, COL5A1
rno04142:Lysosome	4.46E-06	GNS, LAMP1, PSAP, IGF2R, HEXA, LGMN, HEXB, CTSA, CTSB, CLTC, MAN2B1, CD63
rno05222:Small cell lung cancer	8.08E-06	COL4A2, LAMA4, COL4A1, LAMB2, LAMA5, ITGA3, LAMC1, LAMB1, ITGB1, FN1
rno04611:Platelet activation	2.61E-04	ACTB, TLN1, FERMT3, COL3A1, RAP1A, GNAS, ITGB1, COL5A2, COL11A1, COL5A1
rno05144:Malaria	3.07E-04	LRP1, CD81, THBS1, HBB, THBS3, TGFB2, THBS4
rno05205:Proteoglycans in cancer	3.08E-04	ACTB, WNT10B, EZR, WNT3A, RRAS2, MSN, CD63, THBS1, ITGB1, FLNA, TGFB2, FN1
rno05145:Toxoplasmosis	4.35E-04	HSPA1L, LAMA4, LAMB2, LAMA5, LAMC1, LAMB1, ITGB1, HSPA8, TGFB2
rno04145:Phagosome	8.92E-04	ACTB, LAMP1, C3, TUBB5, C1R, TUBA1A, DYNC1H1, THBS1, ITGB1, THBS3, THBS4
rno04610:Complement and coagulation cascades	9.01E-04	KNG1, A2M, MASP1, C3, F2, SERPINC1, C1R
rno05200:Pathways in cancer	0.00132333	HSP90AB1, COL4A2, WNT10B, COL4A1, WNT3A, ITGA3, ITGB1, CXCL12, TGFB2, LAMA4, LAMB2, LAMA5, GNAS, LAMC1, LAMB1, FN1
rno04810:Regulation of actin cytoskeleton	0.00215551	ACTB, EZR, GSN, RRAS2, F2, CYFIP1, ITGA3, MSN, ITGB1, VCL, FN1
rno04670:Leukocyte transendothelial migration	0.00239506	ACTB, EZR, RAP1A, MSN, CXCL12, ITGB1, VCL, THY1
rno05100:Bacterial invasion	0.00880956	ACTB, CLTC, ITGB1, SEPT8, VCL, FN1

of epithelial cells		
rno00010:Glycolysis / Gluconeogenesis	0.02416132	ALDOA, LDHB, LDHA, PKLR, PGK1
rno05206:MicroRNAs in cancer	0.02507586	EZR, TNC, WNT3A, VIM, TNN, THBS1, TGFB2
rno04390:Hippo signaling pathway	0.03342593	ACTB, YWHAZ, WNT10B, YWHAH, WNT3A, YWHAE, TGFB2
rno00511:Other glycan degradation	0.03530391	HEXA, HEXB, MAN2B1
rno04961:Endocrine and other factor-regulated calcium reabsorption	0.03737753	AP2A1, ATP1A1, GNAS, CLTC
rno03050:Proteasome	0.03737753	PSMA1, PSMA6, PSMD2, PSMD3
rno00531:Glycosaminoglycan degradation	0.04248402	GNS, HEXA, HEXB
rno05414:Dilated cardiomyopathy	0.04307507	ACTB, ITGA3, GNAS, ITGB1, TGFB2
rno04015:Rap1 signaling pathway	0.05382354	ACTB, TLN1, FLT1, RAP1A, GNAS, THBS1, ITGB1, EPHA2
rno05134:Legionellosis	0.06031612	HSPA1L, VCP, C3, HSPA8
rno04144:Endocytosis	0.06251678	HSPA1L, TSG101, AP2A1, IGF2R, ARF3, PDCD6IP, CLTC, HSPA8, EHD4
rno04612:Antigen processing and presentation	0.06638948	HSPA1L, HSP90AB1, LGMN, CTSB, HSPA8
rno05020:Prion diseases	0.09466802	NCAM1, HSPA5, LAMC1

Table S4 The number of live and dead cells in the ELVs-collagen gel.

		Total cell number		Dead cells		%Dead cells	
		Control	ELV-H1	Control	ELV-H1	Control	ELV-H1
Day 1	Group1	401	477	12	1	2.992519	0.209644
	Group2	406	375	10	2	2.463054	0.533333
	Group3	532	464	14	1	2.631579	0.215517
Day 3	Group1	547	542	27	4	4.936015	0.738007
	Group2	483	477	22	3	4.554865	0.628931
	Group3	527	493	29	4	5.502846	0.811359
Day 5	Group1	679	507	66	7	9.720177	1.380671
	Group2	623	703	60	9	9.630819	1.280228
	Group3	731	537	70	6	9.575923	1.117318
